# The cassette exons of α-synuclein play distinct roles in membrane remodelling and membrane-mediated amyloid formation

**DOI:** 10.1039/d6sc07425a

**Published:** 2026-11-02

**Authors:** Katherine M. Dewison, Pijush Chakraborty, David J. Brockwell, Sheena E. Radford

**Affiliations:** a Astbury Centre for Structural Molecular Biology, School of Molecular and Cellular Biology, Faculty of Health and Life Sciences, University of Leeds Leeds LS2 9JT UK

## Abstract

Membranes are central to both the physiological and pathological roles of α-synuclein (αSyn). Here, we define the roles of exons 3 and 5 of αSyn in membrane interactions and determine how alternative splicing influences membrane binding, protein conformation, membrane remodelling and lipid-induced amyloid formation. We show that deletion of residues encoded by exon 3 reduces membrane-bound helicity to a greater extent than expected from the loss of those residues alone, diminishes the ability of αSyn to lower the membrane gel-to-fluid transition temperature (*T*_m_), and shifts membrane remodelling behaviour from tubulation to fission. Conversely, deletion of exon 5-encoded residues has little effect on membrane affinity, membrane-induced helix formation, modulation of liposomal *T*_m_, or membrane remodelling activity. Despite this, deletions of these residues play a key role in determining the flux through different pathways for lipid-induced amyloid formation, resulting in the formation of structurally distinct fibril populations. Thus, exons 3 and 5 make mechanistically distinct contributions to the relationship between αSyn and membranes with different effects on protein conformation and aggregation. Together, these findings demonstrate that alternative splicing remodels the interplay between αSyn, membranes, and amyloid formation. Mapping the sequence determinants that underlie these processes provides insight into how alternative splicing changes both the physiological functions of αSyn and its involvement in disease.

## Abstract 2

Membranes are central to both the physiological and pathological roles of α-synuclein (αSyn).

## Graphical abstract

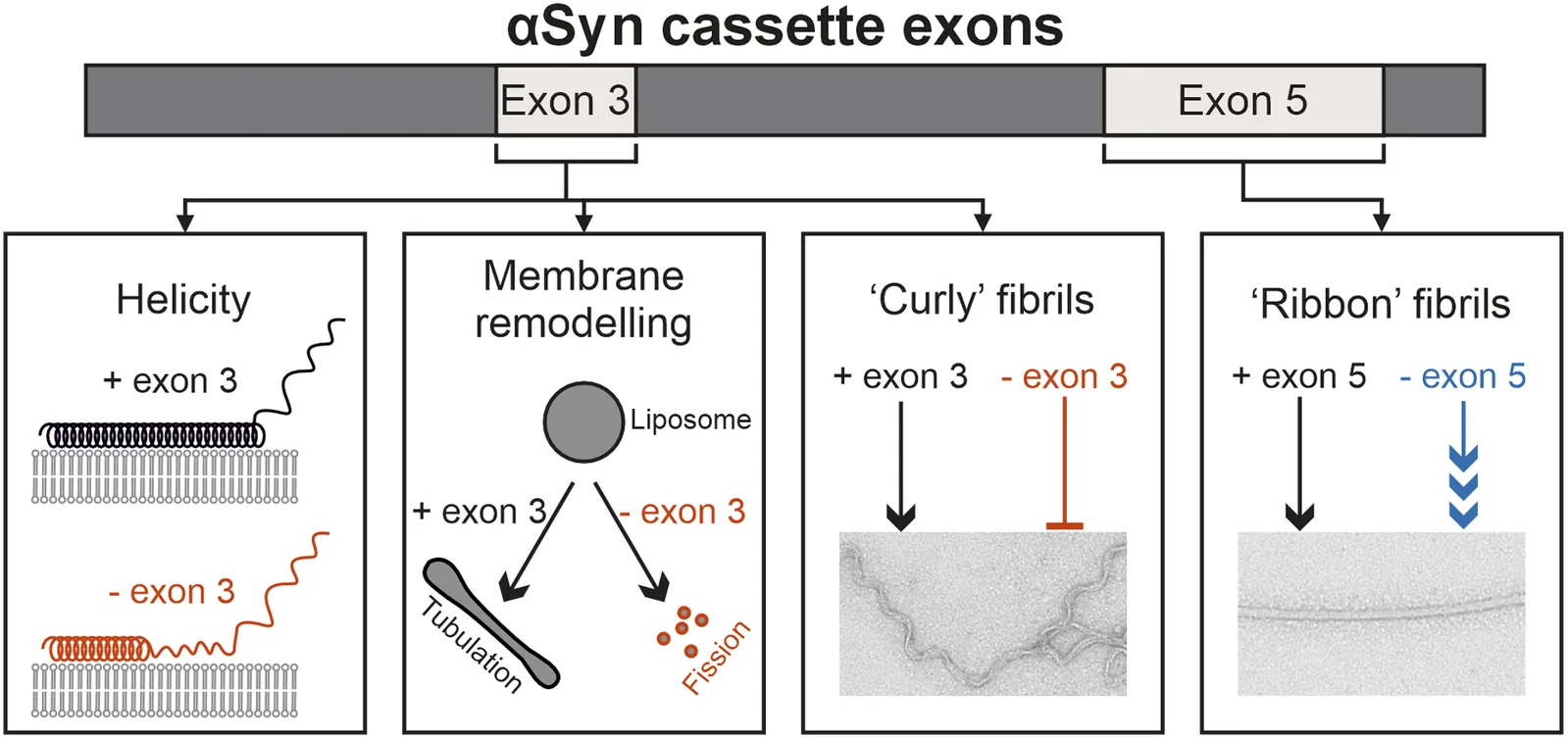

## Introduction

Synucleinopathies, including Parkinson's disease (PD), are hallmarked by the presence of pathological amyloid aggregates containing α-synuclein (αSyn).^1^ The incidence of PD is predominantly sporadic, with only 15% of cases being attributed to familial mutations.^2^ Despite decades of research, the self-assembly mechanisms and the pathogenic species of αSyn relevant to disease remain unclear,^3^ with recent evidence highlighting the importance of lipid membranes in these processes.^4–18^

While recombinant αSyn is intrinsically disordered in aqueous solution,^19^ lipid-binding induces a disorder-to-helical transition in the N-terminal ∼100 residues of the 140 amino acid protein.^20–22^ Membrane-binding and helix-formation have also been shown to result in membrane remodelling, which is essential for the physiological functions of αSyn,^23,24^ including in facilitating synaptic vesicle organisation and neurotransmitter release.^25–28^ It has also been reported that αSyn can adopt a helically-folded tetramer structure when extracted from cells even in the absence of lipid membranes.^29^

Membranes have also been implicated in the pathology of synucleinopathies: the levels of specific lipids are altered in disease^16^ and lipids are a major component of pathological inclusions.^14^ Indeed, it has been proposed that Parkinson's disease should be referred to as a lipidopathy rather than a proteinopathy.^15^ Membranes have been shown to facilitate self-assembly of αSyn into amyloid^5–7,9,11,12^ with lipidic density being resolved in the resulting fibril structures by cryoEM.^17,30^ Lipidic amyloid fibrils that are formed in the presence of synthetic membranes or rat brain-derived synaptic vesicles possess a greater efficiency of cell-to-cell spreading and induction of intraneuronal aggregate formation compared with their non-lipidic counterparts, which highlights the importance of understanding αSyn-membrane interactions to decipher how this increases their toxicity.^18,31^ Furthermore, oligomeric and protofibrillar species of αSyn can exert toxicity by permeabilising membranes,^32^ which leads to mitochondrial dysfunction and oxidative stress.^4,8,10^ The relationship between αSyn and membranes is thus a double-edged sword, as interaction with lipids is required for its physiological function, yet also facilitates its self-assembly into pathological aggregates. Understanding how the balance between function and dysfunction of αSyn is regulated and how this breaks down in disease, remains a central challenge in Parkinson's disease and related neurodegenerative disorders.

The amino acid sequence of αSyn is important in regulating its relationship with membranes.^33–36^ The full-length 140-amino acid sequence of αSyn (αSynFL) is composed of three domains: an amphipathic N-terminal domain (residues 1–60); a central amyloidogenic and hydrophobic NAC domain (residues 61–95); and a highly acidic C-terminal domain (residues 96–140). The N-terminal and NAC domains contain seven imperfect 11-residue repeats of a KTKEGV consensus motif,^37,38^ which facilitates formation of helical secondary structure that is coupled to membrane binding.^39^ Many of the familial mutations in αSyn linked to Parkinson's disease are found in the N-terminal region of the sequence, including A30P, E46K, H50Q, G51D, and A53T.^40^ In addition to αSynFL, alternative splice variants have also been identified and shown to be expressed in neurones.^41–45^ These result, *inter alia*, from skipping of exon 3 (αSynΔ3, residues 41–54, also known as αSyn126), exon 5 (αSynΔ5, residues 103–130, also known as αSyn112), or both exons 3 and 5 (αSynΔ3Δ5, also known as αSyn98)^46,47^ (Fig. 1). Notably, exon 3 contains the sites for many of the familial mutations associated with Parkinson's disease and other synucleinopathies, while truncations of residues in the C-terminal region have been shown to significantly enhance the rate of amyloid assembly.^35,48,49^ Exon 5 also contains the residue S129 which is a dominant phosphorylation site that is often used as a biomarker of pathological αSyn.^50^ As this residue is lacking in αSynΔ5 and αSynΔ3Δ5, aggregates of these variants would not be observed in studies that use S129 phosphorylation as a marker of αSyn pathology.

**Fig. 1:**
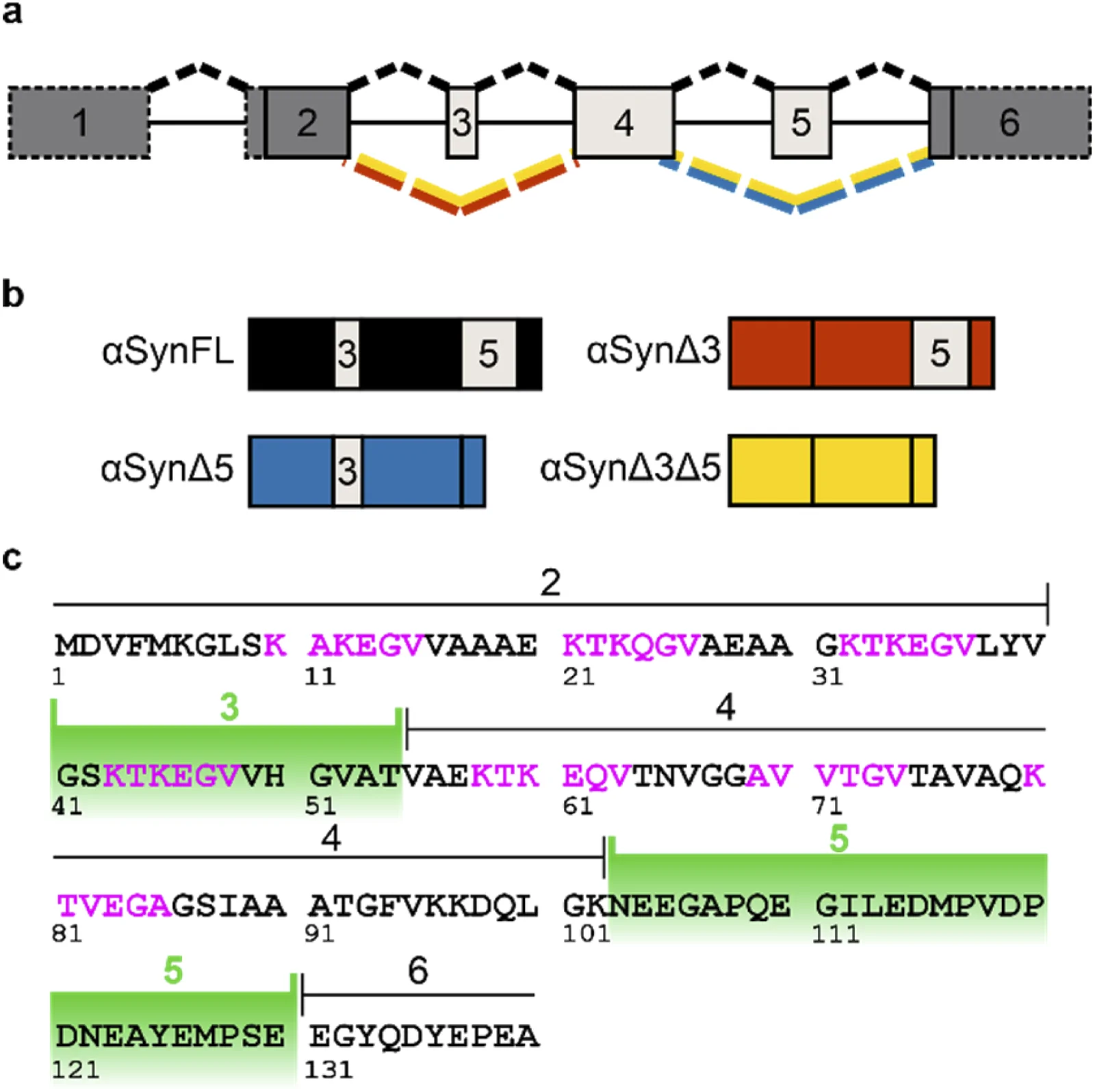
Alternative splice variants of αSyn. (a) Schematic representation of the splicing pathways of *SNCA*. Exons are shown as grey boxes, introns as lines. Boxes with a solid grey line are protein-coding exons. Light grey boxes represent cassette exons, which can be skipped during splicing. The black dashed lines connecting exons represent the splicing pathway of αSynFL, while the coloured dashed lines represent the exons that are skipped for αSynΔ3 (red), αSynΔ5 (blue) and αSynΔ3Δ5 (yellow). (b) Schematic representation of the exons that are included in each of the four splice variants used in this work. (c) Amino acid sequence of αSynFL, with the exon number that each region is encoded by displayed above. The cassette exons 3 and 5 are highlighted in green. The imperfect KTKEGV repeats are highlighted in purple. The figure was adapted from ref. 51.

There has recently been a surge in interest in the effects of alternative splicing in the aggregation potential of αSyn.^22,51–68^ 3–5% of the *SNCA* mRNA transcripts in the frontal cortex encode for splice variants of αSyn^67^ and levels are altered in different synucleinopathies,^41–45,68^ yet even at low levels they have been shown to affect the aggregation of αSynFL into amyloid.^51,55,65,66^ The vast majority of biochemical studies of these proteins have assessed their aggregation potential in the absence of membranes. Specifically, it has been shown that deletion of exon 5 accelerates aggregation into amyloid,^51,55,65^ consistent with previous studies that have shown that other C-terminal truncations increase amyloidogenicity by removal of negative charge.^35,48,49^ Furthermore, mixing just 1% of αSynΔ5 monomers with αSynFL accelerates the nucleation and aggregation of αSynFL,^66^ demonstrating how miniscule concentrations of a protein variant can have significant effects on amyloid formation. By contrast with the behaviour of αSynΔ5, deletion of exon 3 slows amyloid formation under conditions of low ionic strength and results in fibrils unable to cross-seed amyloid formation of αSynFL.^51,65^ These data are consistent with previous findings that the P1 region of αSyn (residues 36–42), which overlaps in part with residues in exon 3, is a ‘master controller’ of amyloid formation.^69,70^

By contrast to these detailed biophysical studies, the membrane interactions of αSyn splice variants have received comparatively little attention and remain poorly characterised and none have explored how alternative splicing affects membrane-catalysed amyloid assembly. Quantification of helicity of αSynΔ3 and αSynΔ5 (named Δ4 and Δ6 in^22^) by circular dichroism (CD) showed that while deletion of exon 5 had little effect, deletion of exon 3 substantially reduced helicity, especially in the presence of phosphatidyl serine (PS) headgroups (αSynΔ3Δ5 was omitted in this study). This leaves open the question of how the naturally expressed αSyn splice variants interact with membranes and whether and how membrane binding catalyses assembly into amyloid.

Here we present the first detailed biophysical analysis of how the different naturally expressed αSyn splice variants αSynΔ3, αSynΔ5, and αSynΔ3Δ5 interact with membranes and how their sequence deletions affect membrane-catalysed amyloid formation. Specifically, we aimed to determine how alternative splicing alters αSyn interactions with liposomes and to assess the consequent effects on both the protein and the lipid bilayer, providing insight into how splicing may modulate αSyn function. We also describe how membrane-binding alters amyloidogenicity across the four protein variants. We describe the membrane-binding affinities and stoichiometries of the splice variants, their membrane-induced helix formation, membrane-remodelling capacity, and the residues involved in membrane interactions using CD, NMR and negative stain electron microscopy (EM). We also compare lipid-mediated amyloid formation across the splice variants by examining the kinetics of aggregation, the morphology of the resulting fibrils, and the fate of the associated lipids. Despite binding to DMPS membranes with similar affinity, the results reveal remarkable and distinct roles of the different exons at every stage of the self-assembly reaction, including the conformational properties of the protein, the effect on lipid dynamics, the mechanisms of amyloid formation, and the morphology of the fibrils formed.

## Experimental

### Materials

1,2-Dimyristoyl-*sn*-glycero-3-phospho-l-serine (DMPS) (105405-50-3) and 1,2-dilauroyl-*sn*-glycero-3-phospho-l-serine (DLPS) (208757-51-1) were purchased from Sigma-Aldrich (Avanti Polar Lipids). 1,6-diphenyl-1,3,5-hexatriene (DPH) (1720-32-7), ammonium sulphate (7783-20-2), Q Sepharose™ Fast Flow (17-0510-01), sodium phosphate dibasic (7558-79-4), sodium phosphate monobasic (7558-80-7), and thioflavin *T* (2390-54-7) were purchased from Merck (Sigma-Aldrich). Carbenicillin disodium (4800-94-6) and isopropyl *β*-d-1-thiogalactopyranoside (IPTG) (367-93-1) were purchased from FORMEDIUM. Ammonium bicarbonate (1066-33-7) was purchased from Fisher Scientific (Thermo Scientific). Chloroform (67-66-3) was purchased from Fisher Scientific (Fisher Chemical). Ammonium chloride (^15^N, 99%) and d-glucose (U-^13^C_6_, 99%) (110187-42-3) were purchased from Goss Scientific. Methanol (67-56-1) was purchased from Fisher Scientific (Acros Organics). Tris Base (77-86-1) was purchased from Fisher Scientific (Fisher BioReagents). Uranyl acetate (541-09-3) was purchased from Electron Microscopy Sciences (EMS).

### Generation of αSyn splice variant expression vectors

Generation of the plasmids expressing the αSyn splice variants was achieved by Q5 site-directed mutagenesis of the pET23a-αSynFL plasmid, as described previously.^51^

### Protein expression and purification

The pET23a plasmids encoding either αSynFL, αSynΔ3, αSynΔ5, or αSynΔ3Δ5 were used to transform *E. coli* BL21 DE3 competent cells by heat shock at 42 °C. The cells were then plated on LB-agar containing 100 µg mL^−1^ carbenicillin and incubated at 37 °C for 16 h. The following day, starter cultures were set up by inoculating 100 mL LB medium (with 100 µg mL^−1^ carbenicillin) with one colony from the LB-agar plate. These were placed in a shaking incubator (37 °C, 200 rpm) for 16 h. The next day, 15 mL of the starter culture was used to inoculate 1 L LB medium with 100 µg mL^−1^ carbenicillin. The flask was then placed at 37 °C, shaking at 200 rpm. When the OD_600_ reached 0.6, 1 mM IPTG was added to the culture to induce expression of αSyn. After incubating for 5 h (37 °C and 200 rpm), the flask was removed from the incubator and cells pelleted at 4000×*g* for 30 min. The cell pellets were stored at −20 °C.

The cell pellets were resuspended in lysis buffer (20 mM Tris HCl, 2 mM MgCl_2_, 5 mM dithiothreitol, 1 mM phenylmethylsulfonyl fluoride, 2 mM benzamidine, 100 µg mL^−1^ lysozyme, 20 µg mL^−1^ deoxyribonuclease I, pH 8), homogenised and heated to 80 °C for 10 min. The samples were then centrifuged at 35 000×*g*. The supernatant containing the αSyn was retained, 30% (w/v) ammonium sulphate was added and the sample incubated on a roller for 30 min at 4 °C. The sample was again centrifuged at 35 000 × g for 30 min and the ammonium sulphate precipitation and subsequent centrifugation step repeated once more. The pellets were combined and stored at −20 °C until further processing.

The pellet was resuspended in buffer for anion exchange chromatography (20 mM Tris HCl, pH 8.0 for αSynFL and αSynΔ3, pH 9.0 for αSynΔ5 and αSynΔ3Δ5). Anion exchange was performed using an 80 mL Q-Sepharose™ Fast Flow column and proteins eluted by increasing the concentration of a second buffer (20 mM Tris HCl, 1 M NaCl, pH 8.0 or 9.0 for αSynFL/αSynΔ3 and αSynΔ5/αSynΔ3Δ5, respectively). The protein in the peak containing αSyn was collected and dialysed into 5 mM ammonium bicarbonate, then lyophilised and stored at −20 °C. Finally, the protein was purified using size exclusion chromatography. The lyophilised protein was resuspended in phosphate-buffered saline (PBS) (137 mM NaCl, 2.7 mM KCl, 8.1 mM Na_2_HPO_4_ and 1.5 mM KH_2_PO_4_; pH 7.4) at approximately 2 mg mL^−1^ and loaded onto a HiLoad® 26/600 Superdex® 75 pg column. Fractions containing αSyn were pooled, dialysed into 5 mM ammonium bicarbonate, lyophilised, and stored at −20 °C.

### Liposome preparation

DMPS (or DLPS) lipids were dissolved in methanol and chloroform at a v/v ratio of 1 : 4, such that 25 mg of lipid was dissolved in 1 mL solvent. The lipids were desiccated using a gentle stream of gaseous N_2_ and vigorous shaking in a 50 °C water bath, until a thin film of lipid was formed. This was placed in a vacuum desiccator overnight. The lipids were then resuspended in 20 mM sodium phosphate buffer, pH 6.5, at a lipid concentration of 40 mM. The lipids were left to rehydrate for 30 min at 50 °C. After this time, the lipids were freeze-thawed five times using liquid N_2_ and a 50 °C water bath. The lipids were then passed through an extruder (Avanti Mini Extruder) set up with a 100 nm filter, which was placed on a heat block at 50 °C. After 21 passes the extruded liposomes were collected.

### Dynamic light scattering

The size and polydispersity of the extruded liposomes were verified using dynamic light scattering (DLS) on a Wyatt miniDAWNTreos®. For this, 20 mM sodium phosphate buffer, pH 6.5 was injected to collect baseline data for approximately five min. After this time, the liposomes (diluted to 8 µM lipid monomer equivalent concentration) were injected and data collected for 5 min. Finally, the buffer was reinjected and data collected for a further five min to determine the baseline. Baseline determination and regularisation was performed using the Astra 3.0.3® software. The distribution of *R*_h_ values was plotted and a Gaussian function was fitted in Graphpad Prism 10.1.2 to determine mean and distribution of *R*_h_ values. The polydispersity index (PDI) of the liposomes was calculated using:1
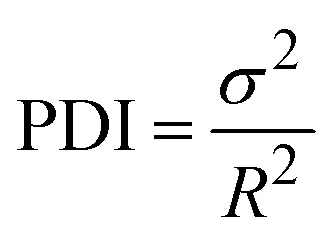
where *σ* is the standard deviation and *R* is the mean radius.

### Circular dichroism

All circular dichroism (CD) spectra were collected using an Applied Photophysics Chirascan spectrometer. Samples were prepared at an αSyn concentration of 25 µM in 20 mM sodium phosphate buffer, pH 6.5 with varying lipid concentrations (lipid-to-protein ratios (LPRs) are indicated in the figures). 1.0 mm pathlength cuvettes were used for spectra collection and the temperature controller incubated the samples at 30 °C. Spectra were collected using a 2.0 nm bandwidth from 260 nm to 185 nm, and the average from three repeat spectra of each individual sample is plotted. The data were blank-corrected and converted from mdeg to mean residue ellipticity (MRE) to account for differences in amino acid sequence lengths:2
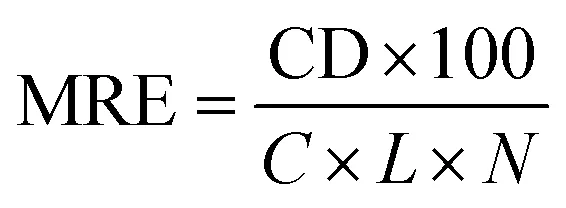
where CD is the measured CD signal (mdeg) at a given wavelength, *C* is the αSyn concentration in µM, *L* is the pathlength of the cuvette (in cm), and *N* is the number of amino acids in the protein sequence.

To determine the binding affinities (*K*_d_) and stoichiometries (*L*) of the αSyn splice variants to DMPS liposomes the following previously-derived^12^ equation was used:3
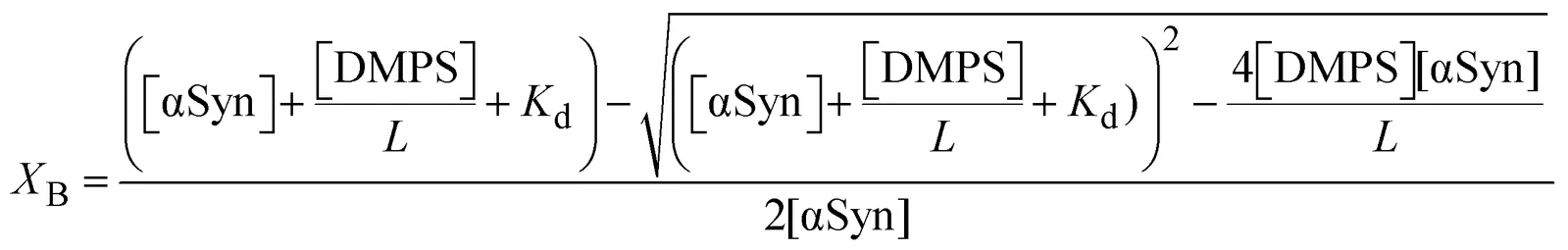
where *X*_B_ describes the fraction of αSyn bound to the liposome and is derived from:4
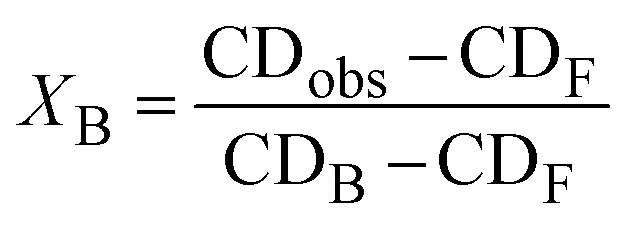
where CD_obs_ is the measured CD signal at 222 nm, and CD_F_ and CD_B_ are the CD signals of the free and 100% bound αSyn, respectively. The CD signal intensity of each αSyn variant in the absence of lipid (CD_F_) was directly measured in experiments using buffer alone (in the absence of liposomes), while the signal intensity of each variant when 100% lipid bound was determined from the intensity of the plateau region at high lipid concentration for each protein (CD_B_).

The percentage of the protein with helical secondary structure^71^ was determined using:5
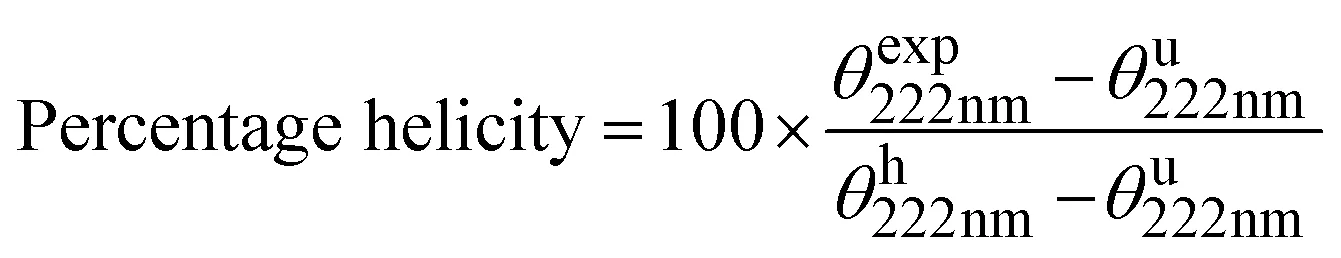
where *θ*^exp^_222nm_ is the experimentally observed helicity under saturating binding conditions. *θ*^u^_222nm_ and *θ*^h^_222nm_ are the estimated values for ellipticity in the disordered (−3000 deg cm^2^ dmole^−1^) and fully helical (−39,500 deg cm^2^ dmole^−1^) states, respectively.

### Nuclear magnetic resonance

2D (^1^H, ^15^N) SOFAST^72^ heteronuclear multiple quantum coherence (HMQC) spectra were recorded at 30 °C using an AVANCE III™ Bruker spectrometer (600 MHz) equipped with a triple channel QCIP cryoprobe. Spectra were recorded on samples of 25 µM [U-^15^N] αSyn in 20 mM sodium phosphate buffer, pH 6.5, 10% (v/v) D_2_O in the absence or presence of DMPS liposomes at an LPR of 8 : 1. All spectra were processed using Topspin 3.7.0 and analysed using CCPN analysis software v2.4.1.^73^ Published assignments of αSynFL (BMRB 16543)^74^ were transferred to the data for the αSyn variants. Peak heights were used to calculate intensity ratios for peaks in the absence and presence of lipid. The CSP was calculated using6
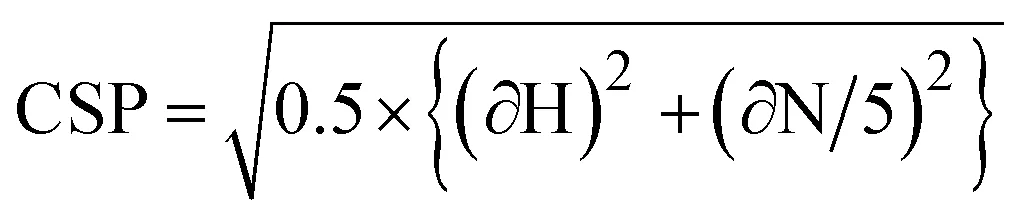


### Membrane fluidity measurements

1,6-Diphenyl-1,3,5-hexatriene (DPH) was used to measure the fluidity of DMPS (or DLPS) liposomes.^75^ DPH was first resuspended in 100% (v/v) ethanol at a concentration of 2 mM. DPH was then added to DMPS liposomes at 600 : 1 ([lipid] : [DPH]) and placed at 45 °C for 30 min to facilitate incorporation of the DPH into the lipid bilayer. αSyn was then added to the lipid-DPH mix at a final protein concentration of 50 µM. The lipid concentration was 400 µM. A PTI Quantamaster Series fluorimeter was used to collect fluorescence measurements at different temperatures with an excitation wavelength of 355 nm, an emission wavelength of 430 nm, at 1 °C intervals. A 2 min equilibration time was used for each measurement. The temperature range used was 15 °C to 50 °C for DMPS and 5 °C to 30 °C for DLPS.

To calculate milli-polarisation (mP), the following equation was used:7
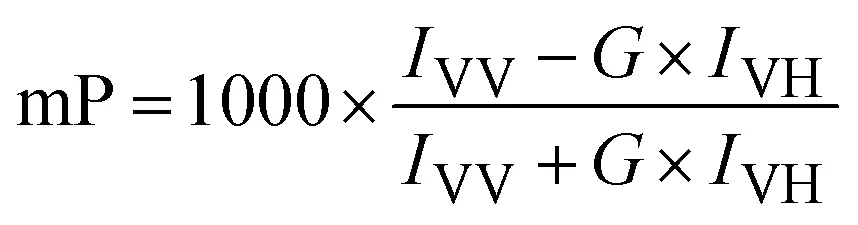
where *I*_VV_ and *I*_VH_ are the measured fluorescence intensities with the emission polarisers in the vertical and horizontal positions, respectively. For both, the excitation polarisers were in the vertical position. *G* is the *G*-factor, which is defined as:8
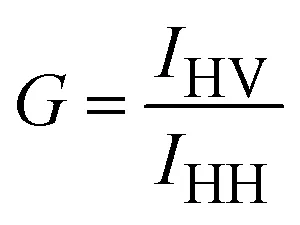
where *I*_HV_ and *I*_HH_ are the measured fluorescence intensities with the excitation polariser in the horizontal position, but the emission polariser in the vertical and horizontal positions, respectively. The first derivative of the data was used to determine the transition temperature of the liposomes in the absence and presence of the different variants of αSyn.

### Negative stain transmission electron microscopy

The presence and morphology of amyloid fibrils and vesicles was examined by negative stain transmission electron microscopy (TEM). For this, 5 µL of sample was applied to glow-discharged carbon-coated grids. After 60 s, the sample was wicked away and the grid was washed three times with 5 µL of H_2_O. 5 µL of 2% (w/v) uranyl acetate was applied to the grid, wicked away, and a further 5 µL of 2% (w/v) uranyl acetate applied again. After 60 s the liquid was wicked away. Grids were imaged using an FEI Tecnai T12 electron microscope.

### Thioflavin *T* assays

To measure the rate of amyloid formation, 50 µM αSyn (in 20 mM sodium phosphate, pH 6.5) was incubated with DMPS liposomes at different LPRs and temperatures as indicated in the figure legends. 20 µM thioflavin *T* (ThT) was used for detection of amyloid formation. 100 µL of sample was added per well of a non-binding 96-well plates (Corning 3651). The plate was incubated in a BMG Labtech FLUOstar Optima plate reader with no shaking. Fluorescence of the wells of the plate was measured using an excitation filter of 440 nm with a bandwidth of 10 nm and fluorescence emission was detected at 480 nm with a bandwidth filter of 10 nm. Three replicates were measured and each experiment was repeated three times.

## Results and discussion

### Residues encoded by exon 3 are required for helix propagation

To determine the capability of the different splice variants of αSyn to bind to model membranes, we chose to use 100 nm synthetic liposomes composed of 1,2-dimyristoyl-*sn*-glycero-3-phospho-l-serine (DMPS) as a model system, as previously used by us and others for αSynFL,^12,36,76^ enabling direct comparison of the behaviour of the three αSyn splice variants with previous reports of the full-length protein. We selected to use protein lacking the natural acetylation at the N-terminus for similar reasons. Whilst other work has utilised sonicated 20 nm liposomes,^13,33^ the difference in liposome size and resulting curvature does not affect the behaviour of αSynFL in this system.^12^ Previous reports comparing N-terminally acetylated and unacetylated αSynFL have shown that acetylation results in a small increase in the helical propensity of residues 1–9 in the absence of lipid,^77–79^ but does not fundamentally alter membrane binding of αSynFL to PC : PS membranes (note that more complex lipid compositions did result in differences in affinity).^78^ In addition, previous studies of N-terminal acetylated and unacetylated versions of the αSyn splice variants studied here has shown that this post-translational modification (PTM) has no effect on the relative rates of amyloid formation of αSynFL and the splice variants in the absence of membranes.^51,55,65^ Liposomes were created by extrusion (SI Fig. 1) and CD used to track the formation of helical secondary structure upon membrane binding.^21,80,81^ The experiments revealed that helical structure is induced by interaction of all four variants with these membranes (Fig. 2). By performing this experiment over a range of lipid-to-protein ratios (LPRs) (0 : 1 to 100 : 1 [M] : [M]) we determined the binding affinity and stoichiometry of each protein variant for the membrane, as described previously^12^ (Table 1). The results showed that all four variants bind to the DMPS liposomes with similar affinity (*K*_ds_ of ∼1 µM), consistent with values obtained for αSynFL reported previously. A similar number of lipid molecules are involved in the interaction for all of the protein variants (Table 1).^12,36^

**Fig. 2:**
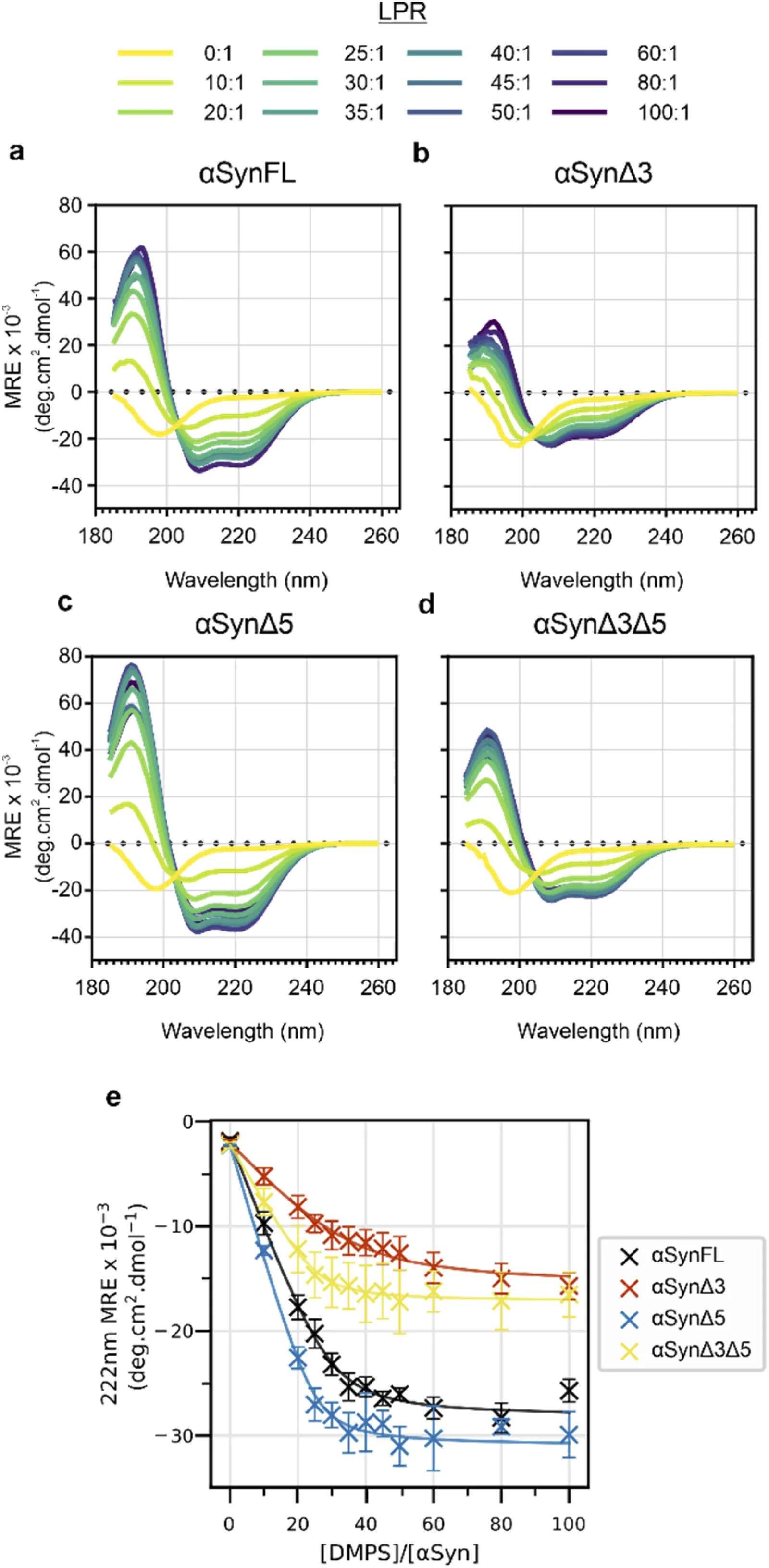
Secondary structure of the alternative splice variants over a range of LPRs. (a) Far-UV CD spectra of αSynFL at LPRs ranging from 0 : 1 to 100 : 1 ([M] : [M]) DMPS liposomes in 20 mM sodium phosphate buffer, pH 6.5, at 30 °C. (b–d), as for (a) for αSynΔ3, αSynΔ5, and αSynΔ3Δ5, respectively. (e) MRE at 222 nm of each of the splice variants across all LPRs. Error bars indicate the standard error of the mean (SEM). Data are fitted to eqn (3) (Experimental).

**Table 1:** Binding affinity, stoichiometry, and maximum helicity of the binding of alternative splice variants of αSyn to DMPS liposomes determined using CD. *K*_d_ and *L* are calculated from fitting eqn (3) (Experimental). Error is SEM. Helicity is determined using eqn (5) (Experimental), and residues in a helix calculated by multiplying the percent helicity by the number of residues in the variant

Variant	*K*_d_ (µM)	*L*	Percent helicity (%)	Residues in helix
αSynFL	1.2 ± 0.4	30 ± 2	69	97
αSynΔ3	3.2 ± 1.2	36 ± 4	35	44
αSynΔ5	0.8 ± 0.3	25 ± 2	77	85
αSynΔ3Δ5	1.0 ± 0.3	26 ± 1	39	38

Although all variants form helical structure in the presence of liposomes, the extent of helix formation differs dramatically for the different proteins. Consistent with literature precedents,^19–21^ ∼100 residues of αSynFL convert to helical secondary structure in the presence of liposomes (69% of residues). For αSynΔ5 ∼85 residues become helical (77% of the sequence). However, in marked contrast, only ∼44 and 38 residues in αSynΔ3 and αSynΔ3Δ5 (just 35% and 39% of the sequence, respectively) convert to a helical form (Table 1). A reduction in lipid-induced helicity has been reported previously for three of the disease-associated familial mutants of αSyn, A30P, G51D, and K58N^33,82^ and also for the rationally-designed αSynΔP1 variant (lacking residues 36–42).^36,69^ Since residues 1–25 of αSyn have been shown to be sufficient to nucleate helix formation in αSynFL,^83^ our data suggest that the residues encoded by exon 3 (residues 41–54) are crucial in facilitating propagation of the helix throughout the N-terminal and NAC domains. By contrast residues in exon 5 have little influence in lipid binding-induced helix formation. These results mirror those obtained previously using small unilamellar vesicles (SUVs) formed from a 1 : 1 mixture of POPA/POPC or POPS/POPC.^22^ Fractionation has also been used to show that deletion of exon 5 does not affect αSyn binding to anionic similar to αSynFL (other variants were not tested).^61^

### αSyn splice variant-membrane interactions visualised using NMR

To identify which residues interact with the lipid membrane interface, ^1^H-^15^N SOFAST heteronuclear multiple quantum coherence (HMQC) NMR experiments were recorded for αSynFL and each of the splice variants in the absence or presence of DMPS liposomes (LPR = 8 : 1) (Experimental). In the absence of lipids, sharp cross-peaks were observed for all residues for each αSyn variant, consistent with their intrinsic disorder (Fig. 3a). Upon addition of DMPS liposomes the intensity of different cross-peaks for each of the αSyn variants was strongly attenuated (Table 2). No chemical shift perturbations (CSPs) or new cross-peaks were detected (SI Fig. 2).

**Fig. 3:**
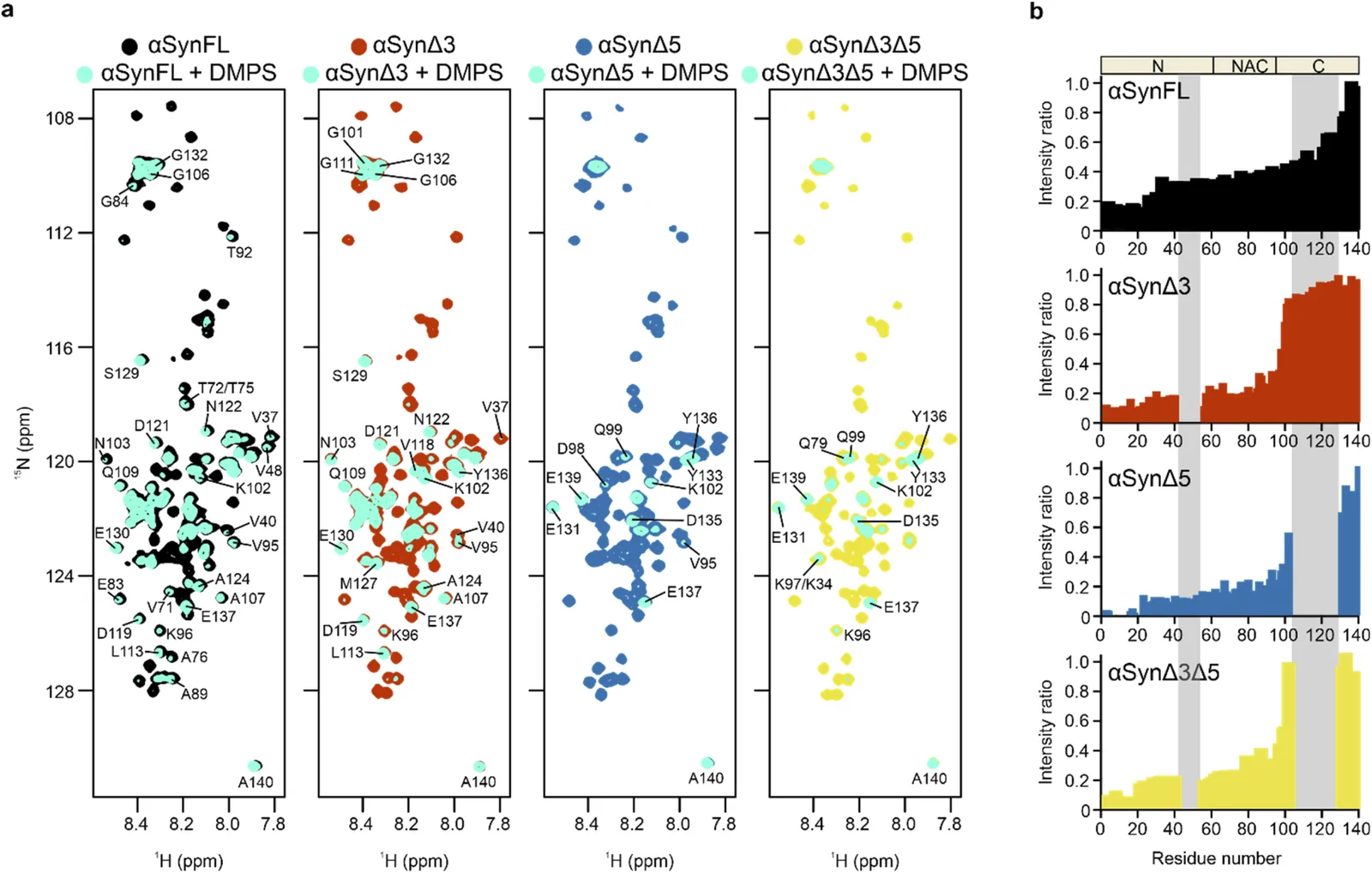
Residues involved in binding to DMPS liposomes. (a) 15N-^1^H SOFAST-HMQC NMR in the absence and presence of DMPS liposomes (LPR = 8 : 1). Spectra were collected at 30 °C. Select residues are labelled in the spectra. (b) Intensity ratio profiles for the αSyn splice variants. Peak heights in the absence and presence of DMPS liposomes were used to determine the intensity ratios. Grey bars indicate regions deleted in the different splice variants.

**Table 2:** Mean intensity ratios for residues 1 to 25, 26 to 99, and 100 to 140 for the alternative splice variants in the absence and presence of DMPS liposomes (LPR 8 : 1) at 30 °C, as measured by ^1^H-^15^N SOFAST-HMQC NMR. Error is standard deviation

Variant	*I*/*I*_0_ res. 1–25	*I*/*I*_0_ res. 26–99	*I*/*I*_0_ res. 96–140
αSynFL	0.13 ± 0.02	0.29 ± 0.05	0.54 ± 0.18
αSynΔ3	0.10 ± 0.02	0.20 ± 0.07	0.87 ± 0.07
αSynΔ5	0.03 ± 0.04	0.15 ± 0.06	0.72 ± 0.20
αSynΔ3Δ5	0.07 ± 0.05	0.22 ± 0.08	0.84 ± 0.15

As shown by Bax and colleagues, lipid binding to αSynFL^84^ and three early onset familial variants of the full-length protein^74^ does not induce chemical shift changes, but instead results in severely decreased signal intensity for N-terminal residues (∼1–25) with a gradual decrease of attenuation towards the C-terminus over a wide range of LPRs (2–173 : 1). This was interpreted as indicating the presence of multiple tightly bound binding modes of αSyn to membranes in slow exchange with each other and the lipid-free state. The ratio of peak intensities in the lipid-bound *versus* lipid-free states for individual residues of αSynFL and the splice variants thus provides a quantitative measure of the fraction of protein molecules for which any given residue has become NMR-invisible due to lipid binding. In agreement with these previous reports,^74,84^ three distinct behaviours of resonances are observed when αSynFL binds DMPS liposomes (Fig. 3b): the N-terminal ∼25 residues are attenuated by ∼85%, residues 26–99 are stronger in intensity (∼70% attenuated), whilst residues 96–140 show gradually increasing intensity, with residues ∼130–140 being unaffected by liposome binding (Fig. 3b (top)). This indicates the existence of at least two distinct pools of lipid-bound protein states that are in slow exchange on the NMR timescale: one in which the N-terminal ∼25 residues are bound, and another in which approximately residues 1–100 are liposome-bound.

For αSynΔ3, αSynΔ5, and αSynΔ3Δ5, the pattern of behaviour observed by NMR differs significantly from that observed for αSynFL, with stronger signal attenuation occurring for their lipid-bound states that spans residues 1–100 (Fig. 3b). This suggests a larger fraction of NMR-invisible, lipid-bound states for all of the variants at this LPR. Furthermore, all splice variants exhibited a sharp increase in intensity ratio for resonances C-terminal to residue 100, in contrast to the more gradual increase observed for αSynFL, suggestive of distinct monomer conformation/dynamics in their C-terminal regions. As the CD spectra of αSynΔ3 and αSynΔ3Δ5 indicate that approximately 40 residues adopt a helical conformation in the liposome-bound state (Fig. 2), and knowledge that helix formation nucleates *via* residues 1–25,^83^ we speculate that although residues 1–100 are lipid-bound as indicated by the attenuation in their NMR resonance intensity, only the first ∼40 residues form a stable α-helix, with residues 40–100 being bound to the lipid surface in a non-helical conformation, perhaps facilitated by electrostatic interactions between positively-charged lysine residues (*i.e.*, K58/K60/K80/K96/K97) and the negatively-charged headgroups of the DMPS lipid. By contrast, for αSynΔ5 most of the lipid-bound sequence (residues ∼1–85) is helical in the membrane-bound state.

### Residues encoded by exon 3 control membrane remodelling

Binding of αSynFL to synthetic membranes (including DMPS liposomes) has been shown to result in membrane-remodelling and transformation of liposomes from their initial spherical state into tubulated forms.^23,24,36,85^ This property is thought to be integral for the function of αSyn at pre-synaptic termini.^23,24^ To determine whether the αSyn splice variants also possess the ability to remodel DMPS liposomes into tubulated vesicles, we incubated each of the variants with DMPS liposomes for 45 h (LPR of 60 : 1), under which conditions amyloid formation does not occur.^12^ The morphology of the liposomes was then visualised by negative stain transmission electron microscopy (TEM). The results revealed liposome tubulation upon incubation with αSynFL and αSynΔ5, while the variants lacking exon 3 (αSynΔ3 and αSynΔ3Δ5) resulted instead in membrane fission (Fig. 4). Notably, αSynΔ3 has been reported previously to result in less membrane damage detected by attenuated total reflection Fourier transform infrared spectroscopy (ATR-FTIR) using POPC/POPG lipids.^59,60^ We have previously shown similar behaviour (fission rather than tubulation) for the variant, αSynΔP1, which lacks residues 36–42 that partly overlap with residues absent in αSynΔ3.^36,69^ The results presented here thus support the previously proposed double-anchor mechanism for αSyn-mediated membrane fusion^24^ and show that residues in exon 3 are required for this function.

**Fig. 4:**
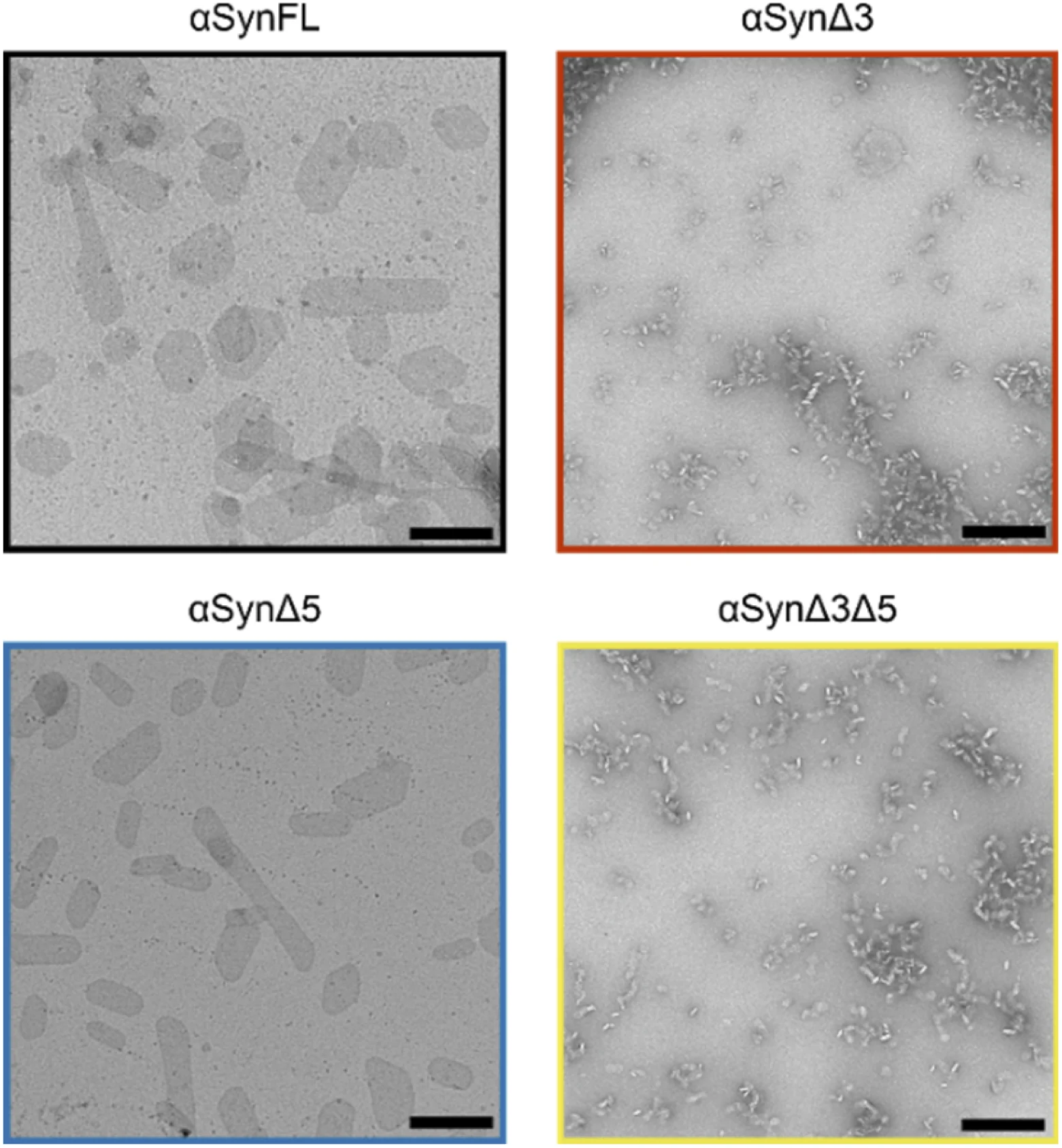
Negative stain TEM images of the DMPS liposomes after 45 h incubation with αSynFL, αSynΔ3, αSynΔ5 or αSynΔ3Δ5 at 30 °C. The LPR was 60 : 1. Scale bar = 250 nm. Note that we have previously reported that drying and staining required for negative stain transmission EM does not impair the integrity of the liposomes used (see Fig. S11 in).^36^

The addition of αSynFL to DMPS liposomes has been shown to lower the lipid gel-to-fluid transition temperature.^13^ To determine whether this is also the case for the splice variants, we measured fluorescence polarisation of 1,6-diphenyl-1,3,5-hexatriene (DPH) to determine how the different interactions of the αSyn splice variants alter the DMPS phase transition temperature of the resulting proteoliposomes (Experimental). Note that in these experiments the readout conflates intrinsic bilayer stability with the effects of protein-insertion and results in an apparent *T*_m_. As expected, all four variants reduced this parameter, with αSynΔ3 having the smallest influence (*T*_m_ = 30.5 °C compared with 37.5 °C in the absence of protein), αSynΔ3Δ5 has a moderate effect (*T*_m_ = 27.5 °C), and αSynFL/αSynΔ5 has the greatest effect (*T*_m_ = 26.5 °C and 25.5 °C, respectively) (SI Fig. 3).

### Membrane-mediated amyloid formation of αSynFL and its splice variants

Incubation of αSynFL with DMPS liposomes at low LPRs has been shown to trigger amyloid assembly, with the maximum rate enhancement occurring at an LPR of 8 : 1.^12^ We next investigated how self-assembly of the splice variants into amyloid is affected by the presence of DMPS liposomes using thioflavin *T* (ThT) fluorescence to monitor amyloid formation (Experimental). Interestingly, while all four variants show membrane-facilitated assembly into amyloid, the rate and amplitude of the final ThT fluorescence intensity was very different for the different proteins (Fig. 5a–c). αSynΔ3 exhibited the slowest rate of amyloid formation (in most cases ThT fluorescence failed to reach a plateau within the timeframe of the experiment). Strikingly, although αSynFL formed ThT-positive assemblies most rapidly (*T*_50_ is 8.1 ± 1.1 h), the ThT fluorescence intensity at 45 h was 10-fold smaller than that of αSynΔ5 or αSynΔ3Δ5, suggesting that fibrils with different structure and hence capacity to bind ThT might result (Fig. 5a–c SI Table 1). To determine whether this is the case, the products formed after 45 h were visualised using negative stain TEM (Fig. 5d, and SI Fig. 4 for further examples). Strikingly, while fibrils were identified for all four variants, the morphology of αSynFL fibrils resembled that reported previously as the ‘protofibril’ or ‘curly’ type,^12,86,87^ while those formed by αSynΔ3, αSynΔ5, or αSynΔ3Δ5 are more similar to ‘mature’ fibrils (straight and ribbon-like). These ‘curly’ and ‘ribbon’ fibril polymorphs are similar to the kinetically-trapped protofibril and mature fibril structures, respectively, described previously for αSynFL formed in the presence of DMPS liposomes.^87^

**Fig. 5:**
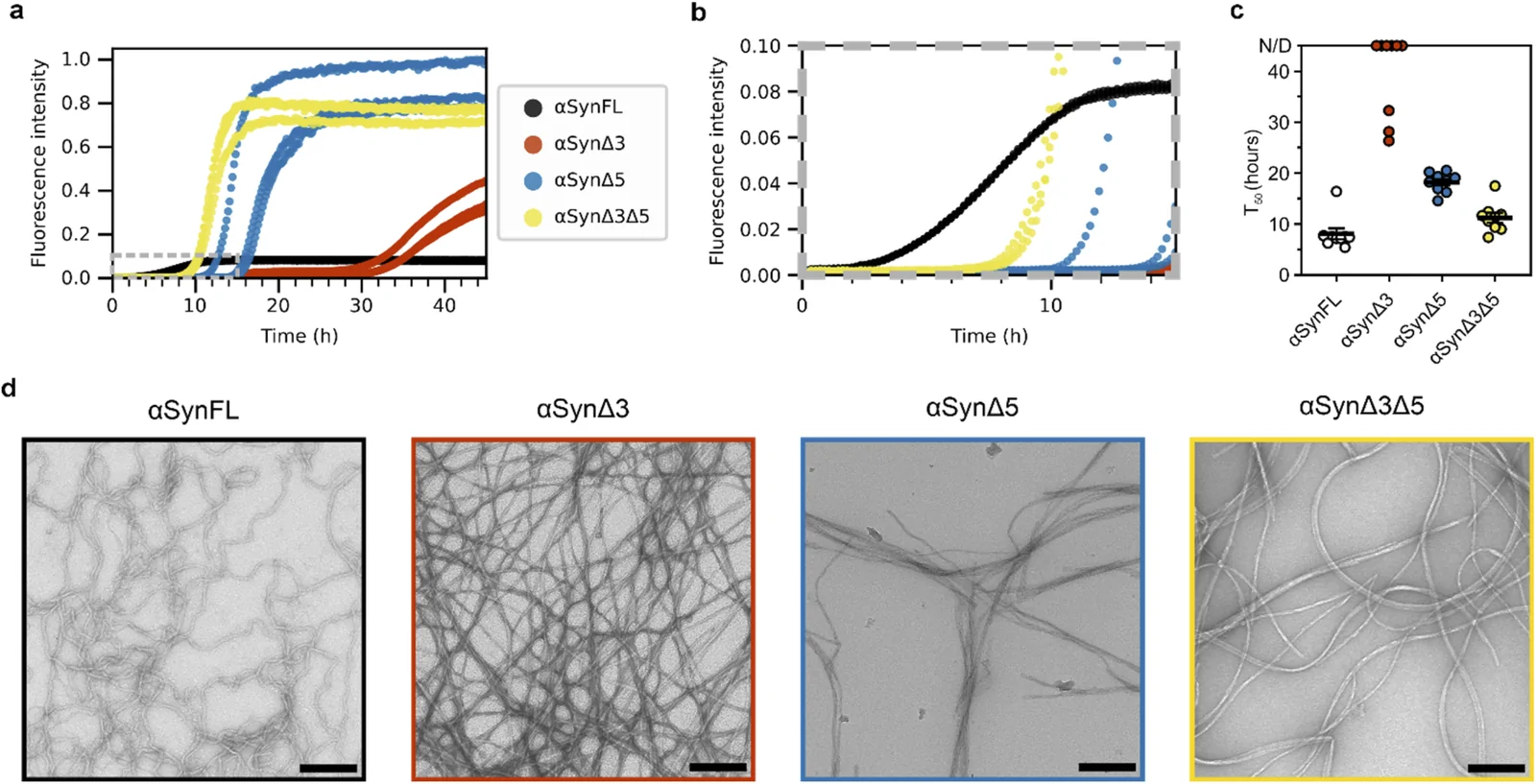
Lipid-induced amyloid formation of the alternative splice variants of αSyn at pH 6.5, 30 °C and an LPR of 8 : 1. (a) ThT fluorescence intensity over 45 h of incubation αSynFL, αSynΔ3, αSynΔ5, and αSynΔ3Δ5 in the presence of DMPS liposomes. The grey dashed line indicates where the data for (b) relates to. (b) Zoom of (a) to show the rate of self-assembly of αSynFL is fastest, but relatively low in ThT intensity. (c) *T*_50_ of fibril assembly. Three repeats from three biological replicates are plotted. The mean and SEM are plotted as lines. Note that in six of the nine repeats for αSynΔ3 the *T*_50_ could not be determined (N/D) as the reaction failed to reach a plateau by the end of the experiment. (d) Negative stain TEM images of the fibrils formed at 45 h. Scale bar is 250 nm. See also SI Fig. 4.

### Unlocking new fibril polymorphs by increased temperature

The protofibrillar (or ‘curly’) state of the αSynFL lipidic-fibrils is known to be metastable in that increasing the temperature can induce transformation into mature ribbon-like fibrils.^87^ To determine whether the fibrils formed by the splice variants are also metastable, despite already appearing ribbon-like, fibrils were again formed by incubation with DMPS liposomes for 45 h at 30 °C. The temperature was then increased to 45 °C and ThT fluorescence continually tracked (Experimental). The results showed, as expected,^87^ that the ‘curly’ αSynFL protofibrils undergo a second growth phase after the temperature jump, forming straight, ribbon-like fibrils with a significant increase in ThT fluorescence (Fig. 6a). Similar to αSynFL (although with differences in total amplitude), the temperature jump also induced a second fibril growth phase for αSynΔ5 and αSynΔ3. By contrast, no further changes were observed for αSynΔ3Δ5 (Fig. 6a). Imaging the samples by negative stain TEM (Fig. 6b) showed the presence of ribbon-like fibrils after the temperature jump for αSynFL, αSynΔ3 and αSynΔ3Δ5. For αSynΔ5, by contrast, transition to a ‘curly’ type polymorph occurred.

**Fig. 6:**
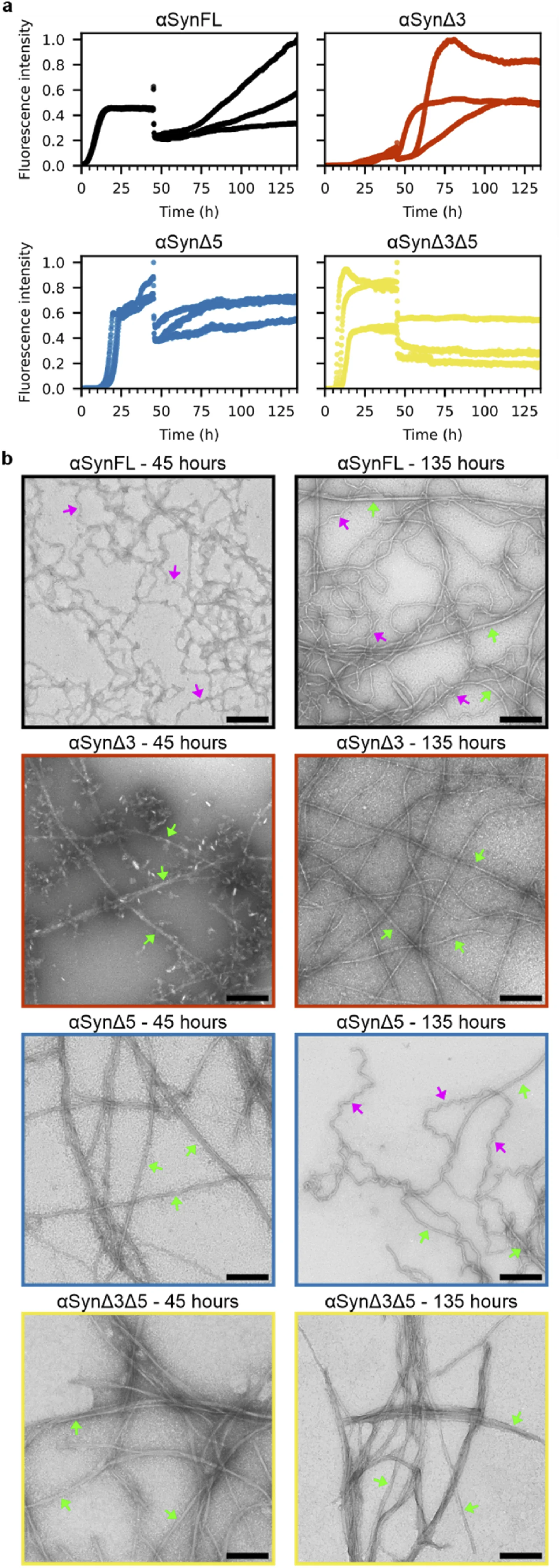
Effect of a temperature jump on the self-assembly kinetics and morphology of fibrils. (a) Lipid-induced amyloid assembly kinetics of the alternative splice variants of αSyn with a temperature jump from 30 °C to 45 °C after 45 h. One representative experiment, containing three repeats is shown. Data are normalised to the maximum intensity detected per variant. Note: the rapid decrease in ThT intensity at 45 h is associated with changing the temperature of the system. (b) Morphology of the different variants after either 45 h or 135 h, imaged by negative stain TEM (as indicated above each image). Green and pink arrows are used to highlight examples of ribbon and curly fibrils, respectively. Scale bar is 250 nm.

The results suggest that the presence of exon 3 and 5 in αSynFL inhibits the formation of ribbon-like fibrils at 30 °C, resulting in curly fibrils that assemble on an alternative assembly pathway, as previously reported.^87^ Destabilisation of these metastable fibrils at higher temperature then enables access to the pathway leading to mature fibrils, with such a transition not being required for sequences that lack one or both exons. Unexpectedly, however, the higher temperature induced the emergence of ‘curly’ fibrils for αSynΔ5, for reasons that remain unclear. Finally, incubation of αSynFL and the three splice variants with DMPS liposomes at 45 °C throughout the reaction (*i.e.* 45 h at 45 °C), leads to the formation of fibrils with a ribbon morphology for αSynΔ3 and αSynΔ3Δ5, but mixed curly and ribbon morphologies for αSynFL and αSynΔ5, highlighting the sensitivity of the energy landscape for membrane-mediated amyloid formation to both the sequence and temperature of the assembly reaction (SI Fig. 5).

### Decreasing membrane stability replicates the effects of temperature

Increasing the lipid acyl chain length has been shown to decrease the rate of amyloid formation of αSynFL,^13^ attributed to the increased thermodynamic stability of the lipid bilayer (*i.e.* the free energy of transfer of the lipid molecule into the bilayer). This suggests that disruption of the bilayer and lipid extraction play an important role in lipid-induced amyloid formation of αSynFL, especially as lipids are integrated into resultant fibrils.^17^ As an increase in temperature destabilises membranes, we investigated how amyloid formation of the splice variants is affected by incubation with a bilayer comprising DLPS (12-carbon acyl chain) at 30 °C, using ThT fluorescence to monitor the fibril growth kinetics and negative stain TEM to image the morphology of the fibrils formed. The results showed that αSynFL forms amyloid fibrils more rapidly in the presence of DLPS liposomes compared with DMPS liposomes (*T*_50_ = 1.3 ± 0.2 h and 8.1 ± 1.1 h, SI Fig. 6), consistent with previous reports.^13^ Of the splice variants, self-assembly of only αSynΔ5 is more rapid in the presence DLPS membrane liposomes (*T*_50_ = 13.8 ± 0.6 and 18.2 ± 0.6 h for DLPS and DMPS liposomes, respectively), while αSynΔ3 assembled slowly, failing in most cases to reach a plateau in the time frame of the experiment. For αSynΔ3Δ5, assembly was slower in the presence of DLPS liposomes (*T*_50_ = 21.7 ± 1.2 h and 11.3 ± 1.0 h for DLPS and DMPS liposomes, respectively). The fibril morphologies accessible to the splice variants in DLPS liposomes were similar to those formed in the presence of DMPS after temperature jump to 45 °C: αSynΔ3 and αSynΔ3Δ5 formed ribbon-like fibrils, while αSynΔ5 formed a mixture of curly and ribbon forms. Together the results highlight the importance of residues in exons 3 and 5 for determining the mechanisms and outcomes of lipid-induced amyloid assembly, at least under the three different experimental conditions tested here.

## Conclusions

Here we describe the first detailed biophysical analysis of the ability of the naturally expressed αSyn splice variants, αSynΔ3, αSynΔ5, and αSynΔ3Δ5, to bind membranes, undergo structural conversion to a helical state, and to induce membrane remodelling, providing insights into how alternative splicing may impact the biological function of αSyn at the pre-synaptic terminal of neurones. In parallel, we used an array of different techniques to explore how these exon deletions affect the rates and structural outcomes of membrane-mediated amyloid formation for these naturally expressed splice variants, of relevance to their impact in disease.

Regarding the importance of alternative splicing in the function of αSyn at a membrane surface, we demonstrate here the crucial requirement of residues 41–54, encoded by exon 3, for facilitating several membrane-associated behaviours of αSyn. We show that deletion of these residues has no effect on the binding affinity of the protein for DMPS liposomes. Despite this, loss of these residues disrupts the propagation of helical secondary structure in the lipid-bound state, reducing the helical content by ∼30% compared with αSynFL despite deletion of just 14 of the 140 residues of the full-length sequence (summarised in Table 3 and Fig. 7a). One reason for this effect could be disruption in the spacing of the highly-conserved KTKEGV motifs caused by this deletion (Fig. 1).^37^ A similar result has been reported previously for αSynΔP1, which lacks residues 36–42 which overlaps, in part, with residues in exon 3.^36,69^ This highlights the potential importance of residues in exon 3 for the physiological function of αSyn at the pre-synaptic termini of neurones which has been proposed to involve a ‘double-anchor’ mechanism that relies on extensive helix formation in the full-length protein.^24^ Notably αSynΔP1, αSynΔ3, and αSynΔ3Δ5 cause fission of DMPS liposomes compared with the fusion observed with αSynFL and αSynΔ5 (Fig. 4).^36^

**Table 3:** Summary of the properties of the alternative splice variants in the presence of liposomes. Unless specified otherwise, the results in the presence of DMPS liposomes are reported

	αSynFL	αSynΔ3	αSynΔ5	αSynΔ3Δ5
*K*_d_	∼1 µM	Similar to FL	Similar to FL	Similar to FL
Helicity	∼100 residues	Reduced	Similar to FL	Reduced
Liposome morphology	Tubes	Fission	Tubes	Fission
Membrane *T*_m_	Low (26.5 °C)	High (30.5 °C)	Lowest (25.5 °C)	Intermediate (27.5 °C)
Residues associated	1–25 tight, 26–99 weak	∼1–99	∼1–99	∼1–99
DMPS amyloid kinetics	Fastest (*T*_50_ = ∼8 h)	Slowest (*T*_50_ > 29 h)	Intermediate (*T*_50_ = ∼18 h)	Fast (*T*_50_ = ∼11 h)
Amyloid morphology	Curly	Ribbon	Ribbon	Ribbon
Temp. jump morphology	Curly + ribbon	Ribbon	Curly + ribbon	Ribbon
45 °C only morphology	Curly + ribbon	Ribbon	Curly + ribbon	Ribbon
Amyloid kinetics (DLPS)	Fastest (*T*_50_ = ∼1 h)	Slowest (*T*_50_ > 33 h)	Intermediate (*T*_50_ = ∼14 h)	Slow (*T*_50_ > 21 h)
Amyloid morphology (DLPS)	Curly	Ribbon	Curly + ribbon	Ribbon

**Fig. 7:**
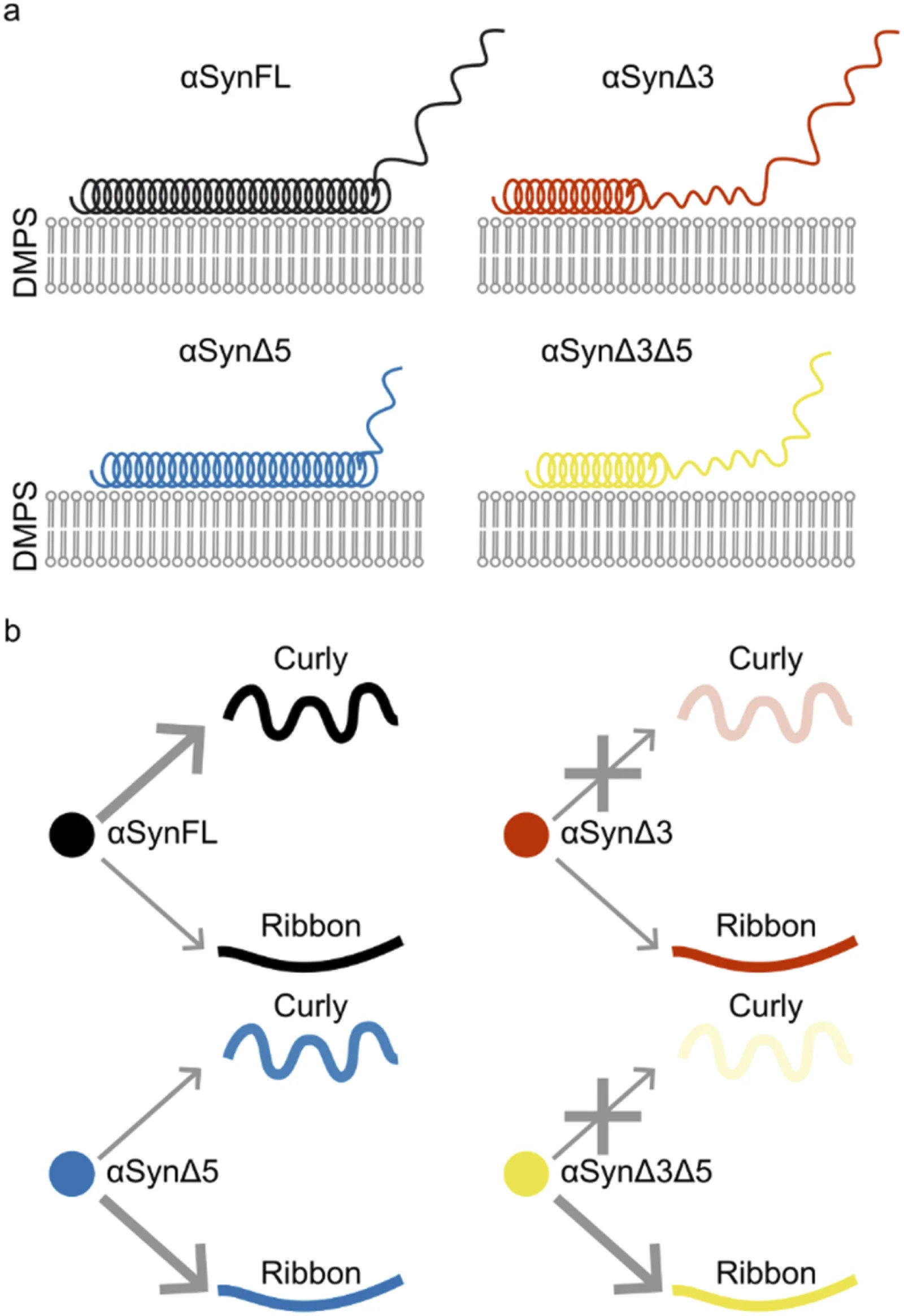
(a) Schematic representation of the helical conformations of the αSyn splice variants on DMPS membranes. Coils and free-drawn lines represent helices and unstructured protein, respectively. Based on the CD and NMR results, the number of residues in the helix and involved in lipid-binding are illustrated. (b) Schematic diagram of the conversion of the αSyn monomers in the different variant sequences into either curly or ribbon fibrils.

It is also important to note that exon 3 contains the sites of several of the familial variants of αSyn^40^ and is also immediately adjacent to Tyr39, phosphorylation of which has been shown to have profound effects both on the function and pathological behaviour of αSyn.^50,88,89^ This modification decreases formation of the membrane-bound helical state and perturbs αSyn's role in synaptic vesicle trafficking and clustering.^90^ It also results in new fibril structures as revealed by cryoEM.^88^ Other PTMs which would not be possible in αSynΔ3 include phosphorylation of S42, acetylation of K43/K45, and the formation of dopamine-derived adducts of K43 which are oriented on the positively charged face of the membrane-induced amphipathic helix of αSynFL.^91–93^ Hence, loss-of-function may contribute to disease in synucleinopathies in neurones expressing this splice variant.

Although lipids are genetically,^94^ histopathologically,^14,15^ and potentially mechanistically^11,12^ central to synucleinopathies, the effects of alternative splicing in membrane-mediated amyloid formation was previously unexplored. Here we show that deletion of residues in exon 3 has a dramatic effect on the rate of fibril formation and the morphology of the fibrils formed compared with αSynFL (Table 3 and Fig. 7b). In the absence of liposomes, deletion of exon 3 has a minimal effect on the rate of amyloid formation,^55,65^ except for low ionic strength conditions under which amyloid assembly slows.^51^ Here, we show that membrane-mediated amyloid assembly is significantly retarded by deletion of exon 3. Most interestingly for αSynΔ3 (and αSynΔ3Δ5) curly fibrils were not observed under any of the experimental conditions tested here, demonstrating that the presence of exon 3 (and the associated behaviours enabled by its residues) is necessary for the protein to form the curly fibril morphology (Fig. 7b). Together, the data presented show that the residues within exon 3 play an intimate role in αSynFL interaction with lipid membranes (at least the DMPS liposomes used here), modulating helicity, membrane remodelling, and reducing the effect on the lipid phase transition temperature compared with αSynFL, αSynΔ5, and αSynΔ3Δ5 (Table 3). As the membrane is extensively remodelled during the formation of lipidic amyloid structures, removal of exon 3 strongly affects the kinetics of amyloid formation in this lipid-dependent pathway.

By contrast with deletion of exon 3, we show here that residues in exon 5 have little effect on membrane-induced helix formation and membrane remodelling (Table 3 and Fig. 7a) using DMPS liposomes. Residues encoded by exon 5 are also heavily post-translationally modified,^50^ and hence these PTMs are not possible in αSynΔ5, and αSynΔ3Δ5. In our study we have used unmodified αSyn proteins and synthetic DMPS liposomes to enable both detailed biophysical analyses and direct comparison with previous studies.^12,35,36,87^ It is possible, therefore, that the effects of deletion of exon 5 may differ in the more complex environment at the synapse, wherein the synaptic vesicles are more complex than those utilised here. Indeed, very recent work by Feng and co-workers suggest that phosphorylation of αSynFL at S129 is functionally important in enabling αSyn to bind to synaptic vesicles in the presence of Ca^2+^.^95^ This would not be possible for αSynΔ5 and αSynΔ3Δ5, which lack this residue.

In the context of amyloid formation, deletion of exon 5 has been shown to enhance the rate of amyloid formation in the absence of membranes,^51,55,65^ consistent with the behaviour of other C-terminal truncations.^35,48,49^ C-terminal truncations of αSyn are also enriched in Lewy bodies, consistent with their enhanced amyloidogenicity.^96^ Here we show that despite having little effect on membrane binding and helix formation, deletion of residues in exon 5 has a dramatic effect on the pathway(s) of membrane-mediated amyloid formation (Table 3). Instead of forming curly fibrils, incubation of αSynΔ5 with DMPS liposomes results in formation of ribbon assemblies at 30 °C, with fibrils of mixed morphology forming at a higher temperature or with DLPS liposomes (Table 3 and Fig. 7b). Hence, our results suggest the presence of at least two kinetically distinct pathways for αSyn fibril formation, with the presence or absence of residues in exons 3 and 5 changing the relative flux through the different pathways, presumably by altering the height of energy barriers of the different routes. Whether the curly fibrils are on-pathway to ribbon-like fibrils or are kinetically trapped off-pathway species cannot be resolved from the data presented here, and more detailed kinetic analysis would be needed to answer this question. Nonetheless, the results presented highlight how protein sequence can tailor the relationship between αSyn and membranes, of consequence both for its function and aggregation, and hence the importance of considering the physiological implications of potential therapeutics targeting the self-assembly of this amyloidogenic protein in disease.

Finally, our results highlight the importance of considering the role of alternative splicing in protein aggregation in amyloid formation in general, even when such variants may form just a minor proportion of total protein synthesised. Given the ubiquity of alterative splicing in the human proteome,^97^ controlling splicing could provide new routes to better understand how and why different proteins for amyloid and cause disease and may contribute to the cell-type vulnerability that is prevalent in amyloid pathologies.

## Author contributions

Conceptualisation: K. M. D., D. J. B., S. E. R.; data curation: K. M. D., P. C.; formal analysis: K. M. D., P. C.; funding acquisition: D. J. B., S. E. R.; investigation: K. M. D., P. C.; methodology: K. M. D., P. C.; project administration: D. J. B., S. E. R.; resources: K. M. D., P. C., D. J. B., S. E. R.; software: K. M. D., P. C.; supervision: D. J. B., S. E. R.; validation: K. M. D., P. C.; visualization: K. M. D., P. C.; writing: K. M. D., P. C., D. J. B., S. E. R.; writing – review & editing: K. M. D., P. C., D. J. B., S. E. R.

## Conflicts of interest

There are no conflicts to declare.

## Supplementary Material

SC-OLF-D6SC07425A-s001

## Data Availability

All data are freely available in a University of Leeds doi: https://doi.org/10.5518/1869. Supplementary information (SI): contains six supplemental figures and one supplementary table. See DOI: https://doi.org/10.1039/d6sc07425a.
