# Supplementary material for: The cassette exons of α-synuclein play distinct roles in membrane remodelling and membrane-mediated amyloid formation
Source: Chem Sci. 2026 Nov 2. Online ahead of print. doi: 10.1039/d6sc07425a (PMC13726718; doi:10.1039/d6sc07425a)
Supplement: Supplementary file 1 [file SC-OLF-D6SC07425A-s001.pdf]

Supplementary Information (SI) for Chemical Science.
This journal is © The Royal Society of Chemistry

Supplementary Information for 'The cassette exons of α-synuclein play distinct roles in membrane remodelling and membrane-mediated amyloid formation'

Katherine M. Dewison[a,b], Pijush Chakraborty[a,c], David J. Brockwell[a*] and Sheena E. Radford[a*]

[a.] Astbury Centre for Structural Molecular Biology, School of Molecular and Cellular Biology, Faculty of Health and Life Sciences, University of Leeds, Leeds LS2 9JT, United Kingdom

[b.] Current address: Interdisciplinary Nanoscience Center (iNANO), Aarhus University, Aarhus C, Denmark.

[c.] Current address: Bind Research, Apex, 1 Tribeca Walk, London, United Kingdom, NW1 0QE

* corresponding authors

s.e.radford@leeds.ac.uk

d.j.brockwell@leeds.ac.uk

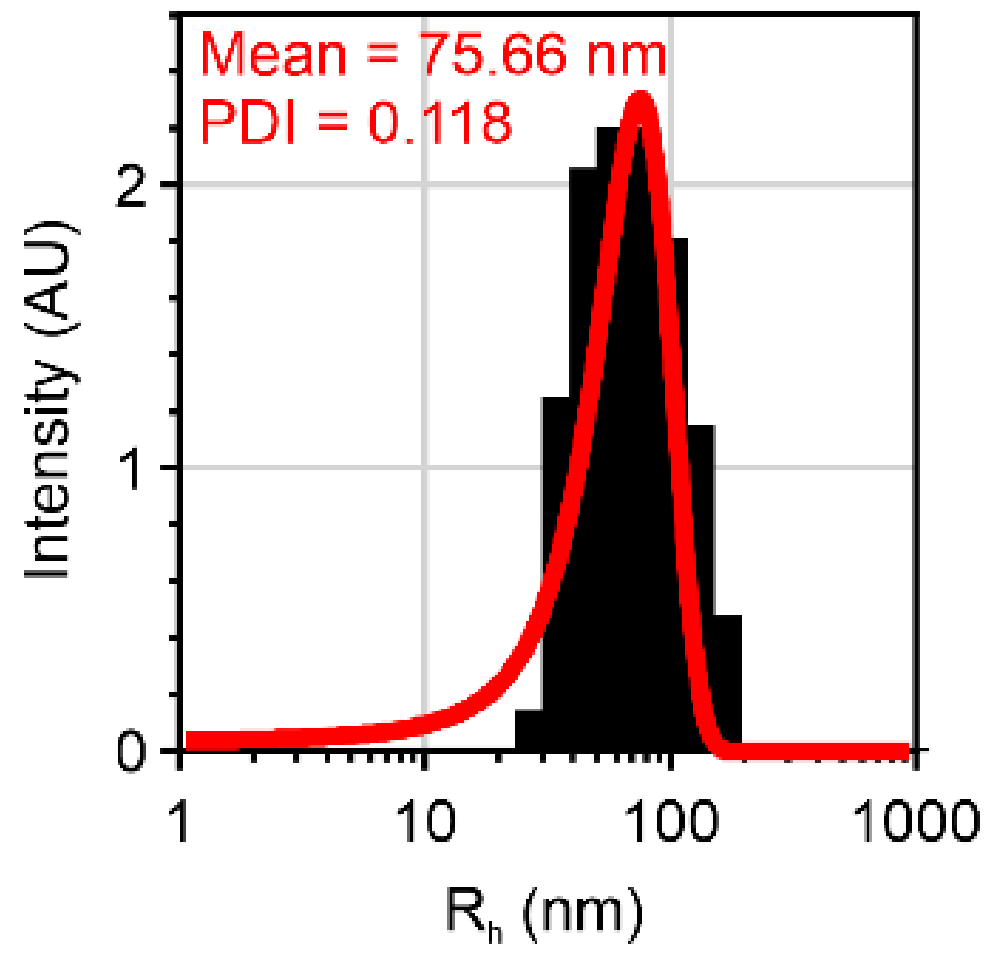

Supplementary Fig. 1. Size and polydispersity of DMPS liposomes measured using dynamic light scattering (DLS)**.** Data are fitted to a Gaussian function (red) to determine the mean radius of the liposomes. Polydispersity index (PDI) of the liposomes was determined using Equation [1] (main text).

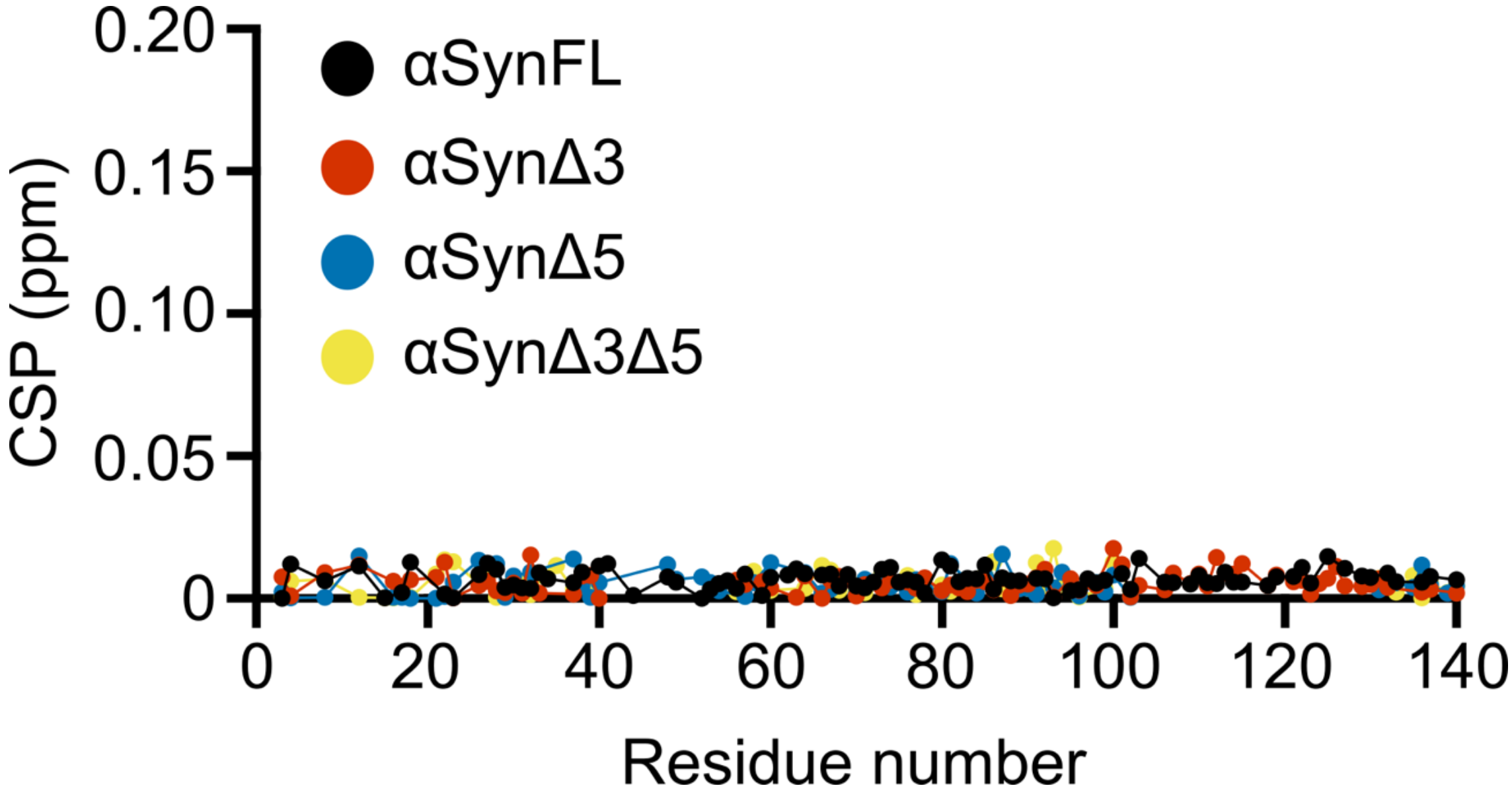

Supplementary Fig. 2. Chemical shift perturbation (CSP) of αSyn splice variants in the presence and absence of DMPS liposomes (LPR = 8:1). CSPs were calculated using Equation [6] (main text).

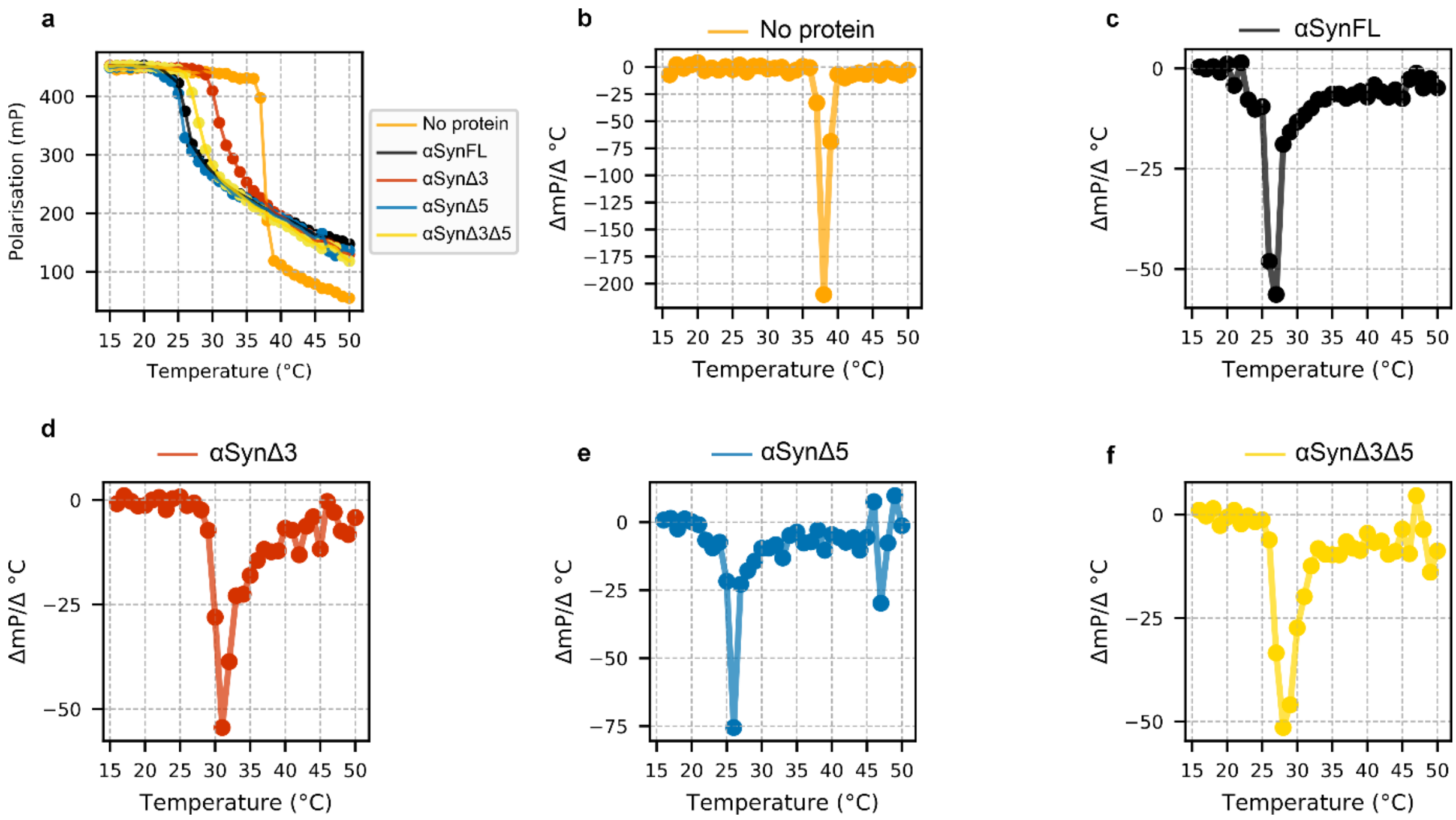

Supplementary Fig. 3. Fluorescence polarisation of DPH-embedded DMPS liposomes in (a) the absence or presence of the different splice variants of αSyn. Polarisation was calculated using Equation [7] (main text). (b-f) First derivative of the data from (a). The phase transition temperatures were determined as 37.5 °C (no protein), 26.5 °C (αSynFL), 30.5 °C (αSynΔ3), 25.5 °C (αSynΔ5) and 27.5 °C (αSynΔ3Δ5).

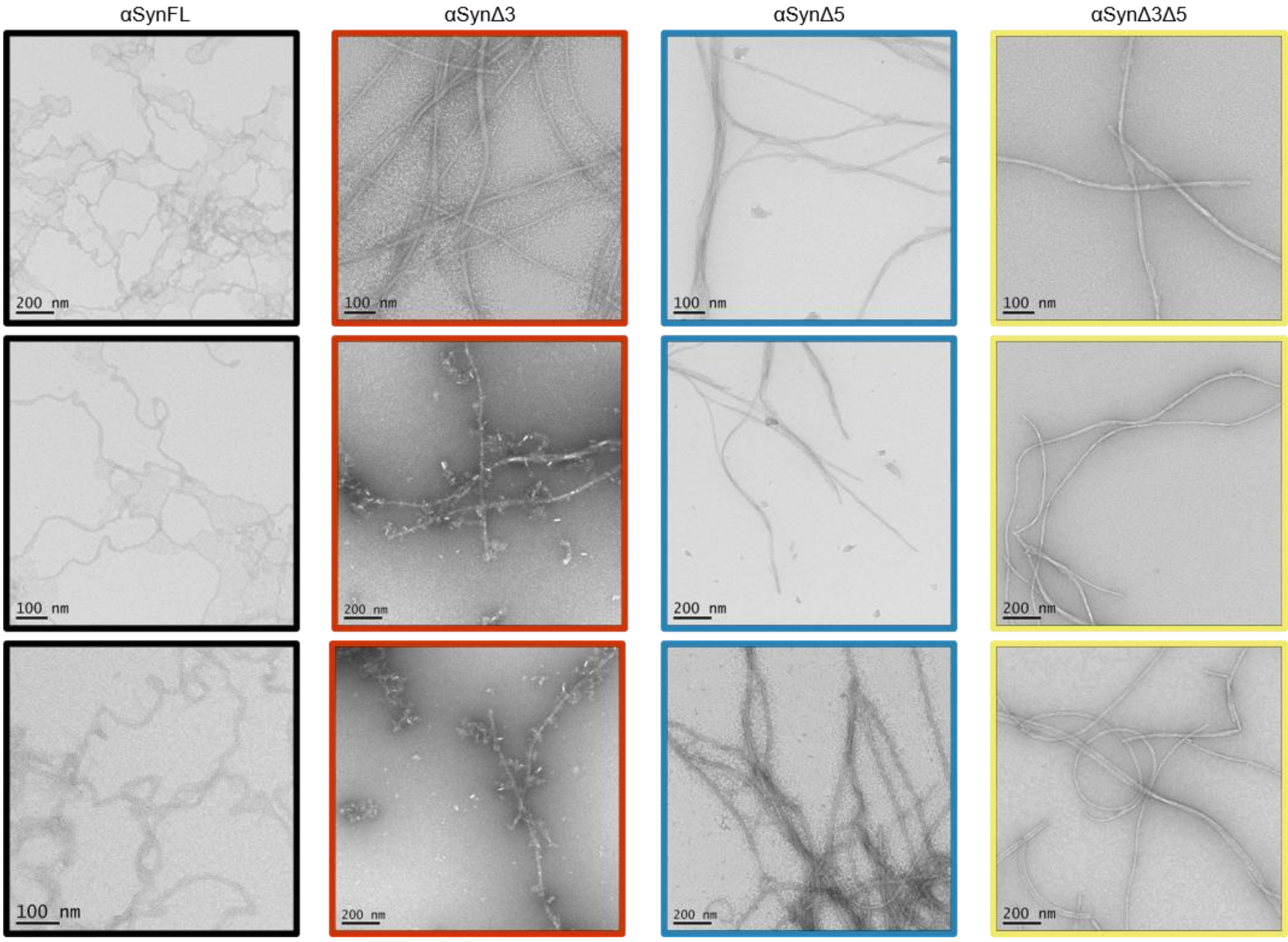

Supplementary Fig. 4. Further examples of negative stain TEM images of lipid-induced fibrils formed from the different proteins after 45 h (pH 6.5, 30 °C and an LPR of 8:1).

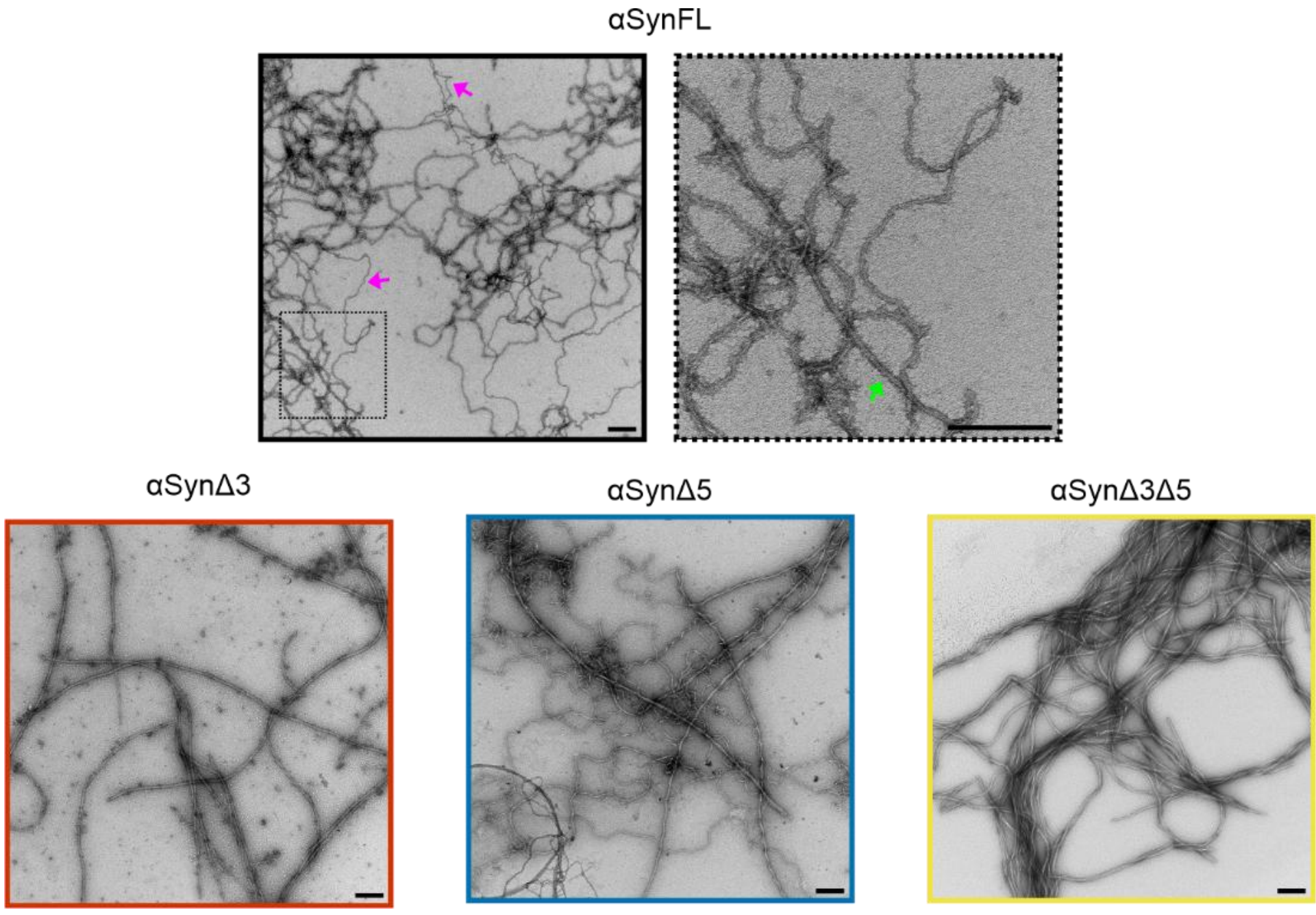

Supplementary Fig. 5. Negative stain TEM images of lipid-induced fibrils formed after 45 h incubation at pH 6.5, 45 °C. Dashed box on αSynFL left image indicates the region of the zoom for the right image. The pink and green arrows point to fibrils with curly and ribbon morphology, respectively. While αSynFL and αSynΔ5 exhibit mixed curly and ribbon fibril morphology, αSynΔ3 and αSynΔ3Δ5 only form the ribbon-like morphology. Scale bar is 250 nm.

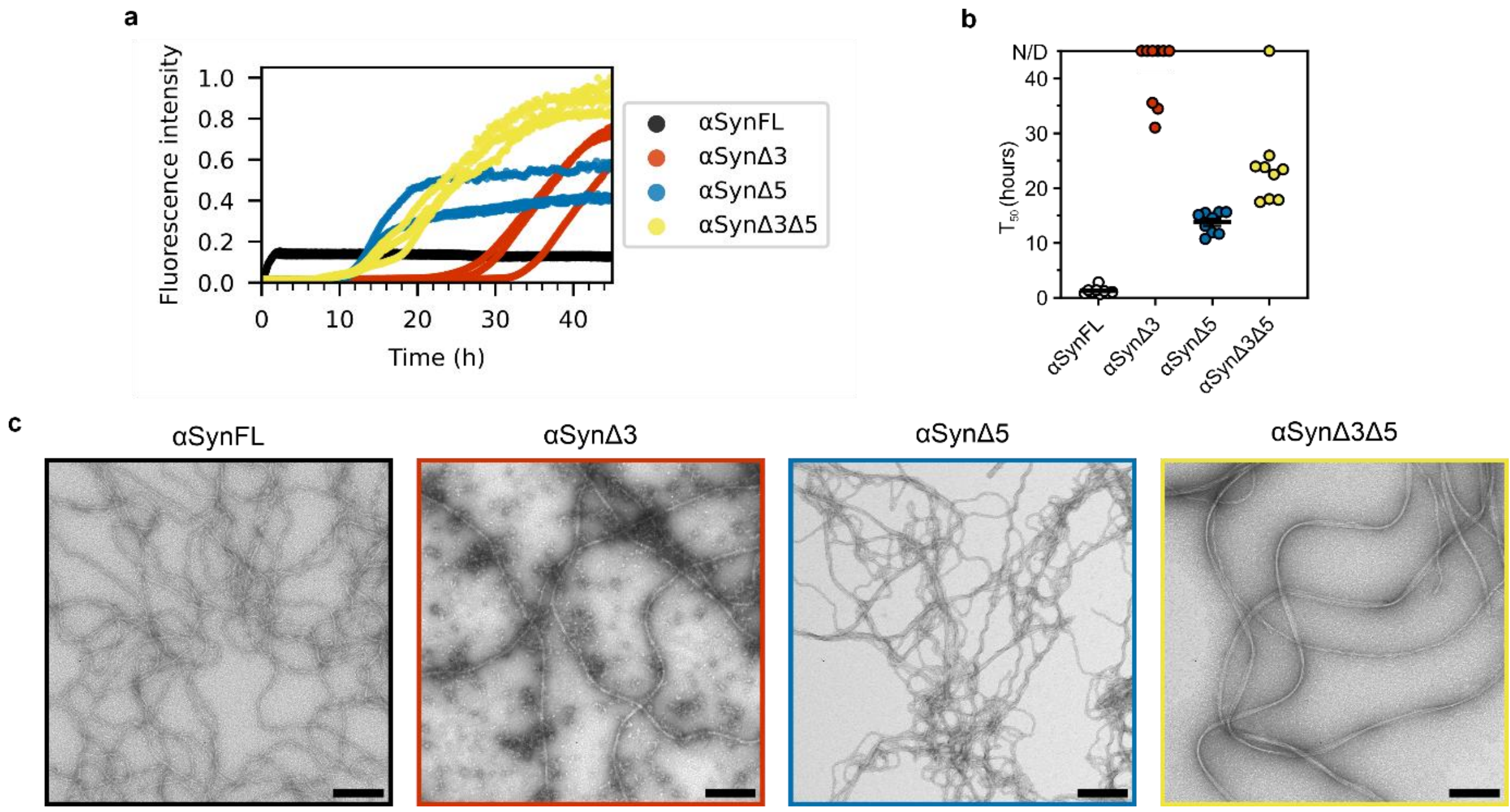

Supplementary Fig. 6. DLPS-induced self-assembly of the alternative splice variants of αSyn at pH 6.5, 30 °C. (a) ThT fluorescence intensity over 45 hours for αSynFL, αSynΔ3, αSynΔ5, and αSynΔ3Δ5 incubated in the presence of DLPS SUVs at a lipid to protein ratio of 8:1. (b) T_{50} values of self-assembly. Note that six of the nine replicates of αSynΔ3 and one replicate of αSynΔ3Δ5 was unable to be determined (N/D) as the fluorescence signal did not plateau over the time frame of the experiment. (c) Representative negative stain TEM images of the samples at the end of the ThT assay. Scale bar is 250 nm.

Supplementary Table 1 Rate of amyloid assembly of the alternative splice variants of αSyn in the presence of DMPS or DLPS SUVs at 30 °C. Error is SEM. Note that these are the means of only the data that plateaued in the timeframe of the experiment.

**Variant**	DMPS T_{50} (hours)	DLPS T_{50} (hours)
αSynFL	8.1 ± 1.1	1.3 ± 0.2
αSynΔ3	29.0 ± 1.8	33.7 ± 1.4
αSynΔ5	18.2 ± 0.6	13.8 ± 0.6
αSynΔ3Δ5	11.3 ± 1.0	21.7 ± 1.2
